# ePRINT: exonuclease assisted mapping of protein-RNA interactions

**DOI:** 10.1186/s13059-024-03271-1

**Published:** 2024-05-28

**Authors:** Sophie Hawkins, Alexandre Mondaini, Seema C. Namboori, Grady G. Nguyen, Gene W. Yeo, Asif Javed, Akshay Bhinge

**Affiliations:** 1 College of Medicine and Health, University of Exeter, Exeter, EX1 2LU UK; 2 Living Systems Institute, University of Exeter, Exeter, EX4 4QD UK; 3 School of Biomedical Sciences, LKS Faculty of Medicine, The University of Hong Kong, Hong Kong SAR, China; 4 Department of Cellular and Molecular Medicine, University of California San Diego, La Jolla, CA USA; 5 Center for RNA Technologies and Therapeutics, UC San Diego, La Jolla, CA USA

**Keywords:** RNA-binding protein, RNA, Regulation, CLIP

## Abstract

RNA-binding proteins (RBPs) regulate key aspects of RNA processing including alternative splicing, mRNA degradation and localization by physically binding RNA molecules. Current methods to map these interactions, such as CLIP, rely on purifying single proteins at a time. Our new method, ePRINT, maps RBP-RNA interaction networks on a global scale without purifying individual RBPs. ePRINT uses exoribonuclease XRN1 to precisely map the 5′ end of the RBP binding site and uncovers direct and indirect targets of an RBP of interest. Importantly, ePRINT can also uncover RBPs that are differentially activated between cell fate transitions, including neural progenitor differentiation into neurons.

**Supplementary Information:**

The online version contains supplementary material available at 10.1186/s13059-024-03271-1.

## Main

**Fig. 1:**
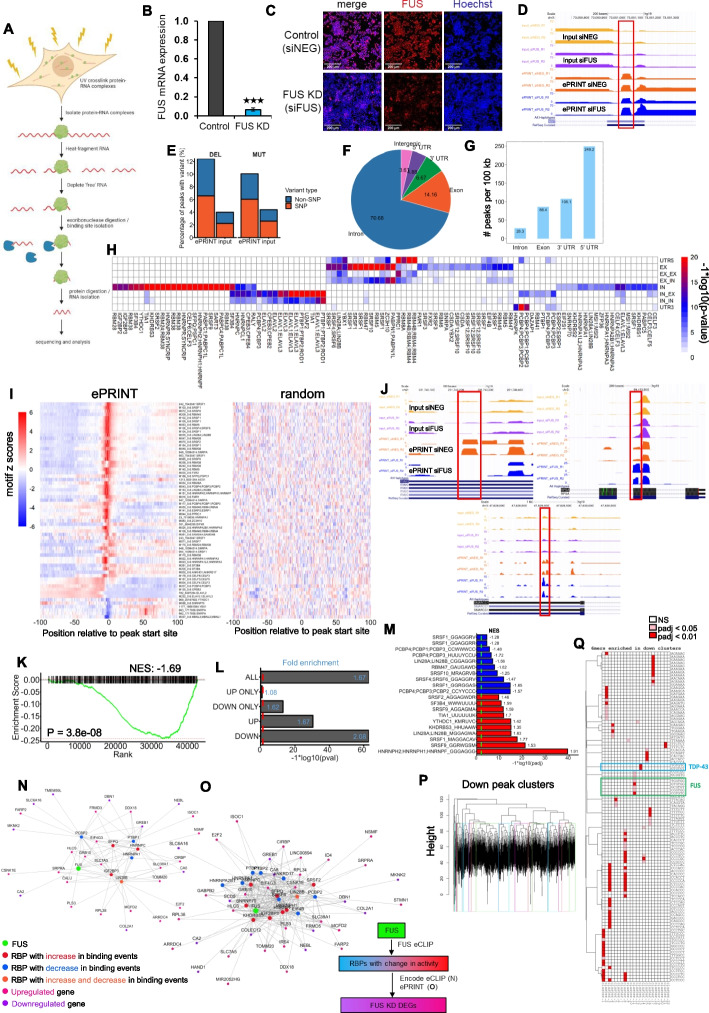
ePRINT identifies bonafide RBP-RNA interactions. **A** Schematic of the ePRINT protocol. Briefly, cells are cross-linked using UV irradiation and then lysed. Protein-RNA complexes are isolated, and then RBP-RNA binding sites are isolated by heat fragmentation followed by 5′–3′ exonuclease digestion. Finally, the protein is digested and the RNA encoding the protein footprint is sequenced. **B** RT-qPCR indicating siRNA-mediated depletion of FUS mRNA in HEK293T cells after 72 h. *N* = 4. *** indicates *p*val < 0.001 by Student’s *t*-test. Error bars indicate SEM. **C** Representative images indicating siRNA-mediated depletion of FUS protein in HEK293T cells after 72 h. **D** UCSC genome browser snapshot showing an example RBP peak in exon 4 of the XIST gene that is enriched in ePRINT vs input, and unchanged between experimental conditions. **E** Mutational analysis showing percentage of peaks with deletions (DEL) or point mutations (MUT). Single nucleotide polymorphisms (SNP) indicate that the mutation/deletion was found within the 1000 genomes database [8]. **F** Distribution of all ePRINT peaks identified in HEK293T cells across the following gene features: introns, exons, 5′ UTRs, 3′ UTRs and intergenic regions. Numbers indicate percentages of ePRINT peaks mapped to a given feature. **G** Number of ePRINT peaks per 100 kb within introns, exons, 5′ UTRs and 3′ UTRs. **H** Enrichment of RBP motifs in peaks mapping to different intragenic gene features. UTR5, EX, IN, and UTR3 indicate peaks where both the start and end sites map within the same 5′ UTR or exon or intron or 3′ UTR, respectively. EX_EX indicates peaks where the start and end sites map to different exons. EX_IN indicates peaks where the start and end sites map to exons and introns respectively. IN_EX indicates peaks where the start and end sites map to introns and exons respectively. IN_IN indicates peaks where the start and end sites map to different introns. **I** RBP motifs are enriched at peak start sites indicated by 0 on the *x*-axis. Peak start sites were extended by 100 bp (+ / −). Scores for individual motifs were estimated at each bp along each peak using the position weight matrices. Per bp scores were averaged across all peaks and converted into *z*-scores: higher z-scores (red) indicate a higher probability of locating the motif(s). The left panel indicates peaks identified in ePRINT samples. The right panel indicates randomly generated peaks. **J** UCSC genome browser snapshots showing example RBP peaks that are altered in the siFUS condition compared to the siNEG control. DOWN/Reduced peak (upper left panel): region: 3′ UTR of the ITM2C gene. Log2FC − 8.64, padj 5.02e − 07. UP/Enhanced peak (upper right panel): region: exon 6 of the RPSA gene. Log2FC 2.47, padj 4.65e − 09. Adjacent UP/DOWN peak (lower panel): region: 3′ UTR of the SMARCC1 gene. UP peak: Log2FC = 1.41. Padj = 5.90e − 13. DOWN peak: Log2FC =  − 0.86. Padj = 4.35e − 04. **K** Peak set enrichment analysis of FUS binding sites identified using eCLIP in HEK293T cells. *X*-axis indicates ePRINT peaks ranked from most significantly upregulated (left side) to most significantly downregulated (right side). A total of 4121 FUS eCLIP peaks mapped to genes considered expressed in the ePRINT analysis; ePRINT captured 25% of these peaks (Fig. S4D). NES indicates normalised enrichment score identified by GSEA. **L** Hypergeometric test to determine enrichment of FUS target genes that display enhanced (UP) or reduced (DOWN) ePRINT peaks upon FUS knockdown. Genes with peaks in both directions are excluded in the UP ONLY and DOWN ONLY comparisons. Values shown in blue indicate fold enrichment of the observed FUS targets in each ePRINT peak group compared to the expected value. **M** Top 10 RBP motifs identified as enriched in peaks that are enhanced (red) or reduced (blue) after FUS knockdown. An enhanced peak indicates that the associated RBP has more binding events, or is more active, after FUS knockdown. A reduced peak indicates that the associated RBP has fewer binding events. NES indicates normalised enrichment score identified by GSEA. **N**, **O** Network analysis to identify direct and indirect effects of FUS knockdown. FUS eCLIP data was used to identify direct targets of FUS. eCLIP datasets (**N**) or ePRINT peaks (**O**) were then used to identify targets of the RBPs showing a change in activity shown in Figs. 1 M and S6B. eCLIP captures 30/53 of the genes that are differentially expressed upon FUS knockdown (DEGs). ePRINT captures 38/53 DEGs. Colour legend on the left indicates expression changes for RBPs and genes. The flow chart on the right displays the strategy used to map the network. **P** Hierarchical clustering of the reduced ePRINT peak set based on sequence similarity. Clusters are indicated by coloured rectangles. **Q** Enrichment of 6-mers in the identified reduced peak clusters. U nucleotides were converted to T to comply with the R alignment package. *P*-values are indicated as not significant (NS), < 0.05 or < 0.01. The green rectangle indicates 6-mers containing the GUGG (GTGG) or GGUG (GGTG) FUS motifs. The blue rectangle indicates 6-mers that are similar to the TDP43 motif (UGUGUG)

Physical interactions between RNA binding proteins (RBPs) and RNAs regulate key aspects of cellular homeostasis including RNA biogenesis, splicing, transport, localization, and decay [1]. Disruption of these regulatory interactions leads to cellular dysfunction and disease [2]. Several RBPs have been implicated in human diseases including cancers and neurodegenerative disorders [3, 4]. Current gold-standard methods to map RBP targets involve biochemical purification of the RBP-bound RNA followed by sequencing of the RNA cargo [5, 6]. This necessitates availability of high-quality antibodies to perform the RBP-RNA purification which excludes a majority of RBPs expressed in mammalian cells. RBPs regulate thousands of genes including those encoding RBPs within intricate interaction networks. Mapping these networks on a global scale requires immunopurification of hundreds of RBPs, a massively expensive and laborious task, especially if the goal is to compare changes in RBP-RNA networks across cell states (for example disease vs healthy). To circumvent these issues, we developed exonuclease-assisted mapping of protein-RNA interactions (ePRINT), a new method that allows mapping RBP-RNA interactions across the transcriptome without the need to purify specific RBPs. Our method exploits the recent observation that organic extraction of UV cross-linked cell lysates causes RBP-RNA complexes to migrate to the interphase [7]. We enrich these RBP-RNA complexes and sequence the RNA to identify the footprint of the bound protein (Fig. 1A). Using bioinformatic analyses, we then uncover the identity of the RBP at each locus and map changes in RBP activity between experimental conditions.

We performed organic extraction on UV-crosslinked HEK293T cells and quantified the amount of RNA retained in the aqueous phase. As expected, treating cells with UV doses of 200 mJ/cm^2^ and 400 mJ/cm^2^ led to a dose-dependent decrease in the levels of aqueous phase RNA, with 400 mJ/cm^2^ recovering ~ 90% of the total RNA from the interphase (Fig. S1A). Having confirmed that covalently bound protein-RNA complexes can be effectively purified, we sought to identify the RNA footprint occupied by the bound protein. We heat fragmented the RNA before organic extraction (Methods) to a median size of 100–200 nucleotides (Fig. S1B) and purified the protein-RNA complexes from the interphase. As expected, crosslinked lysates allowed efficient recovery of fragmented RNA while non-crosslinked lysates showed a significant loss of material (Fig. S1B). However, we observed recovery of low amounts of fragmented RNA from the non-crosslinked samples. To get rid of the observed background, we treated the recovered RNA with T4 polynucleotide kinase to introduce a 5′ phosphate and then digested the repaired RNA with the exonuclease XRN1. We hypothesised that XRN1 activity will cause complete digestion of the unbound RNA while covalently bound protein will physically occlude XRN1 progression, enabling precise mapping of the 5′ end of the protein footprint. XRN1 digestion further reduced RNA amounts from both the non-crosslinked and cross-linked samples with significantly more RNA being recovered from the cross-linked sample (Fig. S1B). However, we were unable to completely remove RNA from the non-crosslinked sample. This could be due to a subset of the RNAs being resistant to XRN1 digestion, possibly due to the 5′ end of the RNAs being protected by a 5′ cap or incomplete repair leading to retention of a 5′ hydroxyl group. Subsequently, we digested the RNA-bound protein with Proteinase K to release the RNA and prepared Illumina-compatible libraries for sequencing using the NEBNext small RNA kit. Our library preparation protocol requires adapter ligation to the 3′ and 5′ ends of the RNA. Hence, any RNA fragments with protected 5′ ends were not expected to be included in the final library. In parallel, we purified 1/10th of each cross-linked sample without performing any enrichment to be used as an RNA fragment size-matched input.

We applied ePRINT to detect global RBP-RNA interaction networks regulated by the RBP FUS. FUS is normally localised to the nucleus and is involved in the regulation of various RNA processing events including alternative splicing, microRNA biogenesis, mRNA stability, transcription, translation and transport, and is also a well-known component of cellular stress granules [9]. It is also found to be dysregulated in multiple cancers as well as neurodegenerative disorders including Amyotrophic Lateral Sclerosis [4, 10]. Given its importance in cellular homeostasis, multiple studies have explored the downstream targets of FUS using CLIP [11–13] making it an attractive choice to validate ePRINT. We knocked down FUS using siRNAs in HEK293T cells (Fig. 1B, C) and performed ePRINT across two biological replicates. We deployed the peak calling software CLIPper [14, 15] to identify RBP binding events. We detected 204,736 peaks that were present in at least two out of the four samples. Analysis of individual binding events across genes showed the importance of using an un-enriched size matched input to filter out background read densities in ePRINT experiments (Fig. S2). We only retained peaks that were significantly enriched relative to the size-matched input and further filtered them based on the peak amplitude and host gene expression (Methods) generating 41,567 high-confidence RBP-RNA binding events (example peak, Fig. 1D).

The identified ePRINT sites overlapped significantly more with at least one ENCODE eCLIP peaks compared to randomly selected sites in the transcriptome (29.84% in ePRINT vs 10% in random simulations, *p*-value < 2.2e − 6) indicating that ePRINT was identifying true binding events. Distribution of the ePRINT peaks across gene features revealed an abundance of sites in introns (Fig. 1F), probably due to their large size relative to other gene features. However, when normalised for the length of each feature, ePRINT peaks had the highest density in the 5′ UTR (Fig. 1G), possibly emphasising the role of RBPs in translational control and mRNA stability. We performed motif enrichment analysis to uncover the identities of the RBPs that potentially bind specific gene features (Fig. 1H). Out of 41,567 binding events, 37,972 were assigned to a known RBP motif. Peaks associated with more than one RBP motif (Fig. S3A) may be indicative of competitive binding, sequence similarity, or localisation of RBP binding sites in close proximity. We found that certain RBP motifs were more promiscuous, such as the RBP KHDRBS3 which is known to bind AU-rich elements (Fig. S3B) [16]. Notably, we also identified RBP motifs that were frequently found together. For example, the motifs M146_0.6 and M062_0.6 have considerable overlap, relating to the RBPs PABPC1 and SART3. This overlap is due to the sequence similarity between these motifs, which both target A-rich sequences. Strikingly, motifs enriched in introns were assigned to RBPs known to function in the nucleus including ELAVL3, SFPQ and several HNRNP proteins, indicating that the identified proteins bind pre-spliced mRNAs in the nucleus (Fig. 1H). These motifs were significantly depleted in other gene features such as exons and UTRs (Fig. S3C). On the other hand, motifs enriched in exons or UTRs belonged to RBPs known to be involved in translational regulation or mRNA localization including LIN28 and FMR1 (Fig. 1H). As expected, such motifs were depleted in the introns indicating these RBPs function in the cytosol (Fig. S3C). Similar results were obtained when the motif enrichments were calculated relative to random peak coordinates selected from within each gene feature (Fig. S3D). We ensured that the random background had 10 times the number of peaks in each feature as compared to ePRINT. Motifs for HNRNPs, CELF3, and SFPQ were enriched in introns while ELAVL3 motifs were enriched at the exon–intron junctions in accordance with our earlier analysis. SRSF and LIN28 motifs were enriched in exons but were also found to be enriched in introns. The differences observed could be because the random simulations were not enriched for regions associated with protein-binding. Mapping motif locations relative to peak start coordinates revealed that most motifs were enriched close to the start site (Fig. 1I). This indicates that XRN1 digestion continues until it is impeded by the protein-RNA crosslinking site allowing precise mapping of the crosslinked location.

Next, we investigated whether ePRINT can identify genome-wide FUS binding sites. We expected that the knockdown of FUS would lead to a decrease in ePRINT peak amplitude at FUS binding sites. Using DESeq2 (Methods), we identified 2753 peaks as increasing in amplitude (enhanced peaks) and 2403 as decreasing in amplitude (reduced peaks) in response to FUS knockdown (example peaks in Fig. 1J). We first identified FUS direct targets in HEK293T cells by performing eCLIP (sequencing statistics included in Table S1). De novo motif analysis of our FUS eCLIP data identified a GGUG/GUGG-containing sequence as the top enriched motif (Fig. S4A), recapitulating previous FUS CLIP results [17]. Additionally, the FUS-associated 5-mers GGGGG and GGUGG identified by RNA-Bind-N-Seq (RBNS) [11, 18] were strongly enriched in our FUS eCLIP peaks (Fig. S4B). Finally, almost 90% of the transcriptome-associated FUS peaks were mapped to introns, as reported previously for FUS binding [19] (Fig. S4C). These results confirmed that our eCLIP data recapitulated FUS binding profiles and was of high quality. We then compared the location of FUS eCLIP binding sites with ePRINT peaks, calculating how close they were to each other. Around 25% of FUS binding sites were within 200 base pairs (bp) of an ePRINT peak, which was significantly higher compared to a random peak set generated within the transcriptome (Fig. S4D). Out of the 4121 high-confidence FUS eCLIP sites identified in HEK293 cells (Methods), only 52 were proximal to at least one of the 4013 enhanced ePRINT peaks (*p*-adjusted < 0.05 and log2 fold change > 1), while 136 FUS sites were proximal to at least one of the 3496 reduced ePRINT peaks detected using the same thresholds (*p*-adjusted < 0.05 and log2 fold change <  − 1). To avoid setting hard thresholds, we used the FUS eCLIP peaks to perform gene set enrichment analysis (GSEA) on our sorted list of ePRINT peaks (most enhanced to most reduced) (Methods). In accordance with our hypothesis, ePRINT peaks that overlapped with FUS eCLIP were significantly represented in the reduced ePRINT peak set (Fig. 1K, Fig. S4E).

We noticed that the number of FUS eCLIP peaks that overlapped with unchanged ePRINT peaks was higher than ePRINT peaks enhanced due to FUS knockdown (Fig. S4E). This could be because an unchanged ePRINT peak might reflect the binding of another RBP to the same site upon FUS knockdown. We did not observe any bias in the enrichment of the RBNS motifs in FUS peaks that overlapped with enhanced, unchanged or reduced ePRINT peaks (Fig. S4E).

We further validated the overlap between ePRINT and eCLIP datasets at the gene level, allowing us to incorporate additional FUS CLIP datasets in our analysis [20–22]. We first mapped all FUS CLIP peaks within intragenic regions to the corresponding gene to define direct FUS targets. Next, we applied the same criteria to differential ePRINT peaks and calculated the overlap between the ePRINT and CLIP gene sets by a hypergeometric test. The overlap was calculated separately for the set of genes that showed enhanced or reduced ePRINT peaks. We noticed that a significant proportion of genes displayed both enhanced and reduced peaks within the gene body. To avoid confounding our results, we also performed the overlap analysis after removing such genes. Gene targets of ePRINT peaks that were reduced after FUS knockdown showed the most significant overlap with FUS eCLIP gene targets (Fig. 1L, Fig. S4F, Additional file 1: eprint vs FUS gene targets), with the enrichments at par when comparing FUS targets across cell lines (Fig. S4G).

Strikingly, the overlap at the gene level returned highly significant p-values compared to the overlap at the peak level. This suggests that FUS has multiple closely aligned binding sites per gene, in accordance with previous observations [23], and each CLIP dataset might be capturing only a subset of these sites. Overall, our analysis indicates that ePRINT can identify FUS binding events across the whole transcriptome with high confidence. De novo motif enrichment analysis on the reduced set of ePRINT peaks identified a GU-rich motif similar to the motif detected in FUS eCLIP (Fig. S4H), that was not detected in the enhanced set of ePRINT peaks. However, the motif was observed in only 5% of the reduced peaks. Previous studies on FUS motif analysis have noted a lack of consistency in assigning a sequence motif to FUS [17]. It is possible that FUS uses a range of motifs for target recognition depending on the cell type.

Based on the available ENCODE data, CLIP experiments typically detect ~ 3000 binding events per RBP (Fig. S5A). HEK293T cells express ~ 1400 RBP proteins [7], indicating we should expect around 4 million binding events total, assuming that each RBP binds independently to its target site and no sites overlap perfectly. Our reported number of binding events (41,567) was much lower than expected, likely due to stringent filtering of the initial set of peaks identified by CLIPper. To ascertain whether the observed results were dependent on the number of peaks detected, we lowered detection thresholds for CLIPper and input thresholding to identify 89,887 intragenic ePRINT peaks in total. Subsequent analysis of motif location and FUS CLIP overlap obtained similar results to our previous thresholds (Fig. S5B–E). Expansion of our peak set resulted in a majority of genes having both peaks that go up and down. This is unsurprising, as transcripts are highly unlikely to be interacting with only a single RBP. This caused a lower number of genes to be included in the DOWN only analysis resulting in higher but still significant *p*-values (Fig. S5E). To keep computation times low, we proceeded with the original set of 41,567 peaks for further analysis.

Since ePRINT, in principle, can capture protein-RNA binding events in an unbiased manner, we used the data to evaluate changes to the RBP-RNA interaction network in response to FUS knockdown. Knockdown of FUS resulted in a change in the distribution of ePRINT peaks across gene features, notably a reduction in the proportion of peaks within exons and 3′ UTRs, and an increase in intronic peaks (Fig. S6A). This suggested compensatory binding of other RBPs to introns, in the absence of FUS. We identified other RBPs that may be affected by FUS knockdown by performing motif analysis on the differentially bound ePRINT peaks (Methods). The reduced set of peaks was found to be enriched for the SRSF RBPs (Fig. 1M, Fig. S6B) in line with the expected role of FUS in regulating alternative splicing. Motif analysis also retrieved the RBP FMR1 (Fig. S6B), which is known to act synergistically with FUS [24]. Accordingly, we observed an enrichment of FMR1 binding sites detected by eCLIP in the set of reduced peaks (Fig. S6C). Our analysis indicates that knockdown of FUS impairs FMR1 binding to cognate RNAs. On the other hand, enhanced peaks were enriched for the HNRNP proteins HNRNPH, HNRNPF, and HNRNPC and TIA1 (Fig. 1M, Fig. S6B). FUS belongs to the HNRNP family, sometimes known as hnRNP P2 [25]. Our analysis indicates that in the absence of FUS, these RBPs might expand their target repertoire to regulate FUS targets, potentially as a compensatory mechanism.

A key limitation of using motif-based analysis for RBP identification is a reliance on the availability of high-quality motif datasets. To address this, we performed GSEA analysis using ENCODE eCLIP peaks as genesets and the sorted set of ePRINT peaks to identify RBPs affected by FUS knockdown. We found 84 RBPs whose direct targets were enriched in reduced peaks (Fig. S6D), and only 1 RBP whose targets were enriched in up peaks (Fig. S6E). Importantly, top candidates identified by the motif analysis as having a reduction in binding events were also identified by CLIP, including the PCBP proteins, SRSF1, FMR1 and LIN28B, while HNRNPC was identified as enriched in the enhanced peaks. RBPs identified using both motif and CLIP-based analysis provide the highest confidence of RBPs with a change in activity. However, this approach will be limited by the availability of such complete datasets. Also, RBPs can have different sets of targets depending on the cell type and species as demonstrated by the overlap of FUS CLIP between cell lines (Fig. S4G). A motif-centric approach offers the advantage of not being dependent on CLIP data available for the specific cell type or species under investigation.

FUS depletion in HEK293T cells resulted in the differential expression of 53 genes (FUS KD DEGs) (Additional file 1: FUS siRNA vs ctrl siRNA DEG). Analysis of our CLIP data suggested that FUS directly interacts with only 8 of these, indicating that the remaining 45 genes could be regulated by RBPs that are downstream from FUS. From the list of RBPs identified from our motif analysis that showed differential binding upon FUS knockdown, we identified 18 RBPs that are direct FUS targets (FUS-RBPs) using FUS CLIP data. Next, we used the ENCODE CLIP datasets to determine which of the 53 FUS KD DEGs are direct targets of our FUS-RBPs. CLIP datasets were available only for 8 out of the 18 FUS-RBPs. Using this approach, we were able to link 30/53 of the FUS KD DEGs to at least one of the FUS-RBPs (Fig. 1N). We repeated this analysis using our ePRINT data to link the FUS KD DEGs to FUS-RBPs. As we were no longer limited by the availability of CLIP datasets, we were able to analyse all candidate RBPs that are directly regulated by FUS. This resulted in linking 38/53 FUS KD DEGs to at least one FUS RBP (Fig. 1O). Importantly, we were able to generate a denser network of the interactions between proteins and RNA within the cell type of interest. Using RBNS motifs instead of the transite motif data in our network analysis led to similar results where we identified 41/53 DEGs (compared to 38 using transite) but a lower number of network edges (244 vs 336) (Fig. S7). An obvious approach to expand the ePRINT-identified networks would be to combine the position-weight matrices with RBNS-identified k-mers to call RBP targets. Thus, using ePRINT, we were able to generate a significantly more comprehensive network of the indirect effects of FUS depletion as compared to using available CLIP datasets (Table S2).

Several RBPs recognise their motifs within structural contexts [26]. We deployed PRIESSTESS [26] to identify motifs that incorporate information on both sequence and structure. We ran PRIESSTESS on ePRINT peaks with enhanced/reduced peaks, and on our FUS eCLIP dataset (Fig. S8). Strikingly, we identified the conventional GUGG motif in unpaired RNA structures in both the FUS eCLIP and ePRINT reduced peaks (Fig. S8A, B). This fits previous observations where FUS was shown to bind stem loop structures [27]. Interestingly, we also observed enrichment of a GAGG motif on RNA loops in both the CLIP and ePRINT reduced peaks (Fig. S8A, B), suggesting that this could also be a widely used FUS motif in HEK293T cells.

Finally, we performed an unbiased clustering analysis to discover novel motifs in our ePRINT peaks (Fig. S9A). We clustered enhanced and reduced peaks separately based on the presence/absence of unique 6mer sequences (Fig. 1P, Fig. S9B). We then performed an enrichment analysis to determine 6mer sequences enriched in particular clusters compared to all ePRINT peaks (Fig. 1Q, Fig. S9C). Using this approach, we were able to identify a single cluster in reduced peaks (cluster 11) that was enriched for GUGG sequences (Fig. 1Q). FUS and TDP-43 are closely related in structure, function and implication in diseases such as Amyotrophic Lateral Sclerosis (ALS) [28]. Using our kmer approach, we identified a small cluster of reduced peaks matching repetitive UG sequences, which is commonly associated with TDP-43 [29] (Fig. 1Q). This could indicate that FUS depletion leads to a reduction in specific TDP-43 binding events. To explore the potential of ePRINT for identifying novel RBPs, we lastly grouped the enriched kmers into known/unknown groups based on presence/absence in the Transite database. We then took the unknown kmers and performed GSEA analysis to confirm enrichment in enhanced or reduced peaks, resulting in a final set of 133 unknown kmers linked to a change in RBP activity (Fig. S9D, Additional file 1: Unknown kmer enrich FUSeprint). Notably, many of these 133 kmers have similar sequences, likely corresponding to only a handful of RBPs. Future experiments combining ePRINT analysis and Bind n Seq assays could identify RBPs linked to these novel motifs [30].

**Fig. 2:**
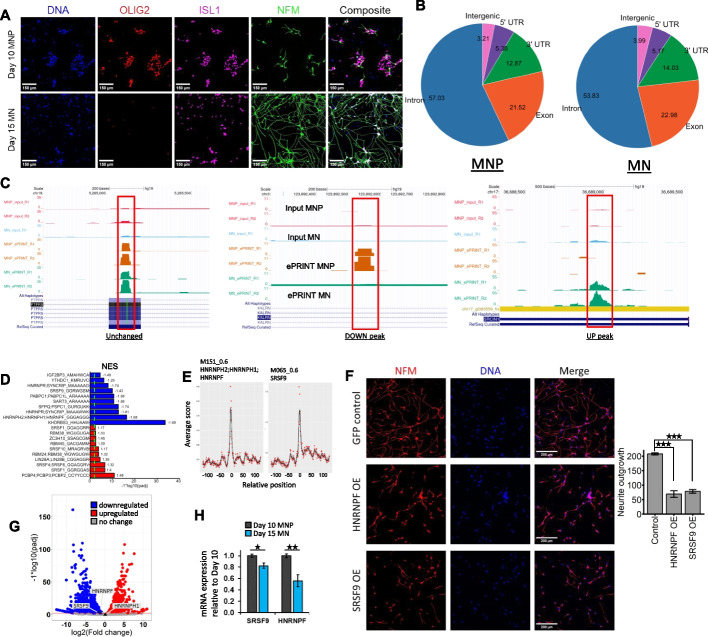
ePRINT uncovers RBPs regulating cell fate transition from motor neuron progenitors (MNPs) to post-mitotic motor neurons (MNs). **A** Representative images of day 10 motor neuron progenitors (MNPs) and day 15 post-mitotic motor neurons (MNs). **B** Distribution of ePRINT peaks across gene features in MNPs vs MNs. Numbers indicate percentages of ePRINT peaks mapped to a given feature. **C** UCSC genome browser snapshots showing example RBP peaks. Unchanged peak (left panel): region: exon 5 of the PTPRS gene. Log2FC 4.86e − 03, padj 1 (0.9999719). DOWN peak (middle panel): Peak that was identified as reduced in MNs compared to MNPs. Region: intron 1 of the KALRN gene. Log2FC − 8.76, padj 8.21e − 05. UP peak (right panel): Peak that was identified as enhanced in MNs compared to MNPs. Region: exon 19 of the SRCIN1 gene. Log2FC 12.45, padj 6.46e − 07. **D** Top 10 RBP motifs identified as enriched in peaks that are enhanced (red) or reduced (blue) in MNs compared to MNPs. NES indicates normalised enrichment score identified by GSEA. **E** Motifs M151_0.6 (HNRNPH2, HNRNPH1, HNRNPF) and M065_0.6 (SRSF9) display enrichment at the peak start site (indicated as 0 on the *x*-axis). Peak start sites were extended by 100 bp on either side for the motif analysis. **F** Representative images of day 16 MNs after RBP overexpression (OE) (left panel) and quantification of total neurite outgrowth normalised by cell count (right panel). Control indicates eGFP expression. Cells were stained with NFM to define the soma and neurites. Nuclei were stained using Hoechst (blue). **G** Volcano plot of differentially expressed genes from day 10 MNP to day 15 MN. SRSF9, HNRNPF and HNRNPH1 have been annotated (light grey labels; triangle points). HNRNPH2 was not expressed in either MNPs or MNs. **H** RT-qPCR analysis of SRSF9 and HNRNPF mRNA expression levels between day 10 MNPs and day 15 MNs. *N* = 3. * indicates *p*val < 0.05. ** indicates *p*val < 0.01. *** indicates pval < 0.001 by Student’s *t*-test. Error bars indicate SEM

Next, we evaluated the efficacy of ePRINT in determining RBP-RNA network changes across cell state transitions. The differentiation of self-renewing stem cells into neural progenitors and post-mitotic neurons is regulated by an intricate network of RBPs [31–33]. We deployed ePRINT to identify key RBPs regulating the transition of motor neuron progenitors (MNPs) into motor neurons (MNs). OLIG2 + MNPs were derived from human iPSCs and further differentiated into OLIG2-/ISL1 + MNs by pharmacologically inhibiting NOTCH signalling [34] (Methods). Immunostaining for OLIG2, ISL1 and NFM indicated that MNPs and MNs were generated at high efficiency using our protocol (Fig. 2A). ePRINT and peak finding was performed as described above with minor modifications (Methods).

We did not observe any significant changes in the distribution of peaks across gene features between the MNP and MN ePRINT samples (Fig. 2B). Differential peak analysis using DESeq2 identified 889 peaks as enriched in MNPs and 5123 peaks enriched in MNs. Example peak changes can be seen in Fig. 2C. Motif analysis on the differentially expressed peaks identified RBP motifs specifically enriched in MNPs or MNs (Fig. 2D, Fig. S10). We mapped motif locations with respect to the peak coordinates and observed an enrichment of the motif around the peak start site for several top candidates (Fig. S11, Fig. S12). We decided to focus our attention on the RBP motifs that displayed a sharp enrichment at the peak start site, and that were predicted to drive MNP self-renewal (Fig. 2E). We hypothesised that overexpression of RBPs responsible for maintaining MNPs in a state of self-renewal would inhibit neuronal differentiation. We delivered constructs encoding the RBPs HNRNPF and SRSF9 to MNPs and then induced them to differentiate using NOTCH inhibition (Fig. S13). Strikingly, over-expression of both candidate RBPs resulted in a significant reduction in neurite outgrowth compared to overexpression of GFP alone (Fig. 2F, Fig. S14). Although both genes show downregulation at the transcript levels from MNP to MN (Fig. 2G, H), neither rank highly in the list of 163 RBPs which are differentially expressed between cell states (Additional file 1: MN vs MNP RBP DEG). This highlights the power of ePRINT in identifying RBPs in terms of their activity and not simply a change in expression between cell states, a feature that will be instrumental for understanding RNA regulation in development, health and disease.

To further ascertain the mechanism behind the observed phenotypes, we sought to identify the downstream targets of these RBPs. To accomplish this, we mapped the motifs of HNRNPF and SRSF9 to ePRINT peaks upregulated in MNPs vs MNs. The identified peaks for each RBPs were then mapped to protein-coding genes that were deemed as downstream targets of the said RBPs. Based on this analysis, we identified 995 peaks mapping to 857 genes for SRSF9 and 695 peaks mapping to 623 genes for HNRNPF. Gene ontology enrichment analysis on these genesets uncovered pathways targeted by these RBPs (Fig. S15). HNRNPF targets were enriched for cell–matrix adhesion, which are highly active in differentiation, embryonic development and remodelling events [35]. SRSF9 targets were enriched for morphological and developmental processes, alongside histone methylation. This suggests that SRSF9 may regulate cell morphology and chromatin accessibility.

ePRINT simultaneously estimates transcriptome-wide RBP binding thereby capturing a comprehensive picture of the role of all RBPs under a given condition and time point. However, that comes with the limitation of reliance on motif-based analytics to tease apart the roles of individual RBPs. In this study, we used 174 motifs mapping to 142 RBPs from the transite database [36]. These numbers are expected to increase with advancements in motif detection and the availability of high-quality CLIP datasets. Our ePRINT analysis is predicated on the hypothesis that changes in RBP target profiles are detectable through differential motif analysis in ePRINT peaks. Such changes could result from alterations in RBP expression, localization, or regulated binding, like needing a specific partner for the target location. ePRINT can identify shifts in RBP target profiles if these RBPs recognise certain motifs. However, motif enrichment does not always imply higher RBP binding. This discrepancy can arise from motifs hidden in secondary structures leading to alternative RBP binding, competitive binding of two RBPs to similar motifs where the RBPs display opposite binding patterns across the two conditions, and if multiple RBPs share similar motifs. Another limitation of our study is the undersampling of binding events. This could be due to the library preparation protocol we used that relies on ligating adapters to both 5′ and 3′ ends of the RNA fragments first and then converting the RNA into cDNA using reverse transcription. Since the reverse transcriptase commonly terminates at the protein-RNA cross-linking site, these fragments will not be enriched by PCR and will be lost. This is likely to lower the sensitivity of our peak detection analysis. Future applications of ePRINT can improve recovery of binding sites by incorporating the eCLIP library protocol [37] and increasing the depth of sequencing. Although the overlap between the reduced ePRINT peaks and FUS eCLIP sites was significant, the extent of the overlap was modest (Fig. S4E). This could be attributed to ePRINT undersampling global binding sites in addition to eCLIP detecting only a subset of the multiple closely aligned FUS binding loci. Additionally, FUS knockdown likely induces significant secondary changes in RBP profiles across the transcriptome that dominate the RBP binding landscape detected by ePRINT.

It must be noted that ePRINT may fail to detect true positives. For instance, MSI1 and MSI2 are RBPs important for the self-renewal of neural stem cells that our study did not identify. This could be because the motifs identified for these RBPs are of low quality. Additionally, the thresholds we used to identify motifs in ePRINT data might not have been ideal. We chose a relative log-odds threshold of 0.8 for identifying motifs, but this might not work best for all motifs, depending on their specificity. Selecting thresholds based on background score distributions could lead to more accurate detection of RBPs [38].

Our method improves on existing methods that also aim to map transcriptome-wide RBP binding events [39–41]. ePRINT does not rely solely on organic extraction or nitrocellulose-based purification to enrich RBP-RNA complexes as this alone is insufficient to deplete unbound RNA. Additionally, the method by Schueler et al. [41], relies on over-digestion of the RNA to get a higher resolution footprint (20–60 nt fragments). This makes mapping the reads to junctions challenging. Our method uses multiple rounds of organic extraction followed by XRN1 digestion to get rid of free RNA thus allowing a much higher signal-to-noise ratio. Additionally, the XRN1 digestion allows mapping the 5′ end of RNA footprint at near single-nucleotide resolution while leaving a longer 3′ end fragment. This allows precise mapping of the RNA fragment across the genome including spliced junctions. Thus, we maintain the resolution of the RBP footprint without compromising our mapping abilities. Further, ePRINT can be applied for post-mortem tissue analysis, as live cells are not a requirement. Our analysis approach enables global identification of RBPs with a change in activity between cell states, whereas previous methods have focused on RBP profiles on a small subset of RNAs in a given cell state. Finally, by mapping ePRINT peaks to gene features, our analysis is accurately able to predict the cellular localisation of RBPs (Fig. 1H, Fig. S3C, Fig. S3D). This could be instrumental in uncovering mislocalisation events often observed in neurodegenerative diseases such as ALS.

In summary, ePRINT can be deployed to identify downstream targets of a single RBP or map changes in the RBP interactome on a global scale as cells transition from one state to another in a cost-effective manner. With the increasing evidence implicating RBPs in a variety of diseases including neurodegeneration and cancer, we expect ePRINT will be a powerful method to understand the mechanistic basis of how RBP networks are altered in diseases.

## Methods

### 293T maintenance

HEK293T cells were maintained in Dulbecco’s modified Eagle’s medium (DMEM; Merck) supplemented with 10% foetal bovine serum (Merck), 2 mM GlutaMAX (Gibco) and 10 mM Hepes (Gibco).

### FUS knockdown

HEK293T’s were seeded at a density of 200,000 cells per 6-well. The next day, cells were transfected with 5 nM Silencer Select siRNA (Thermo Fisher; siNeg, siFUS) using calcium-phosphate (Takara). Media was changed at 24 h and samples were collected 72 h post-transfection.

### Immunofluorescence

Cells were fixed in 4% paraformaldehyde, permeabilized in ice-cold methanol and blocked in 10% serum (HEK293T) or 1% BSA (MN). Primary antibodies (FUS 1:100 Santa Cruz Biotech sc-47711; ISL1 1:500 Abcam ab109517; OLIG2 1:100 R&D Systems AF2418; NFM 1:1000 Merck MAB1621) were added in 1% BSA and incubated at 4 °C overnight. The next day, wells were washed with PBS, then incubated with Alexa-Flour secondary antibodies (Molecular probes; 1:2000) and Hoechst 33,542 (Molecular probes, 1:1000) at RT for 1 h. Cells were imaged using the ImageXpress Pico (Molecular Devices), or DMi8 microscope (Leica). Image quantification was completed using Cellprofiler [42].

### RT-qPCR

Cells were lysed and RNA extracted using the Monarch Total RNA Miniprep Kit (NEB) or RNA cleanup kit (NEB) according to manufacturer instructions. cDNA was reverse transcribed using random hexamers and the High Capacity reverse transcription system from Applied Biosystems. Quantitative PCR was performed using the SYBR GREEN PCR Master Mix from Applied Biosystems and the target gene mRNA expression was normalised to the expression of 2–4 housekeeping genes (HPRT1, RPL13, GAPDH and ACTB). Relative mRNA fold changes were calculated by the ∆∆Ct method. Primer sequences are included:

**Table:** 

Target	Forward 5′ to 3′	Reverse 5′ to 3′
FUS	CAGACAGGGAAACTGGCAAGCT	GGCGAGTAGCAAATGAGACCTTG
HPRT1	CATTATGCTGAGGATTTGGAAAGG	CTTGAGCACACAGAGGGCTACA
RPL13	CCTGGAGGAGAAGAGGAAAGAGA	TTGAGGACCTCTGTGTATTTGTCAA
GAPDH	CAGCCTCAAGATCATCAGCA	TGTGGTCATGAGTCCTTCCA
ACTB	TGACATTAAGGAGAAGCTGTGCTAC	ACTTCATGATGGAGTTGAAGGTAGT
SRSF9	CCTGCGTAAACTGGATGACACC	CCTGCTTTGGTATGGAGAGTCAC
HNRNPF	CTCAGTGATGGCTACGGCTTCA	TGTGGTGCTCTGCACTGTGAAC

### ePRINT

#### UV crosslinking

At least 200,000 cells were washed 1 × with ice-cold PBS (+ 0.49 mM MgCl2, + 0.9 mM CaCl2) and replaced with fresh ice-cold PBS. Cells were irradiated at 400 mJ/cm^−2^ and then cooled by the addition of further ice-cold PBS. Cells were either lysed directly from the plate or gently scraped from the plate and centrifuged at 1200 RPM, 5 min, 4 °C and supernatant discarded. Pellets were stored at − 80 °C.

#### Lysis and protein-RNA isolation (phase separation 1)

Cells were lysed in QIAzol according to manufacturer instructions. A representative sample was isolated for input processing prior to the addition of 1/5th volume of chloroform (200 μL chloroform per 1000 μL QIAzol). The solution was thoroughly mixed and allowed to stand for 2–3 min prior to phase separation at 12,000 g for 15 min at 4 °C. The interphase was isolated and precipitated using 9 × ice-cold methanol, then washed 1 × in 0.3 M guanidine hydrochloride in 95% ethanol, 1 × in 80% ethanol and then allowed to air dry for 5 min. The isolated protein-RNA complexes were then resuspended in a protein resuspension buffer (0.5% SDS, 50 mM Tris–HCl, pH 7.5) for 15–30 min at 60 °C, 300 RPM.

#### Fragmentation

Protein-RNA complexes were fragmented using heat and magnesium (HEK293T cells) or ultrasonic shearing (MNPs and MNs). Briefly, HEK293T samples were diluted in protein resuspension buffer with 10 mM MgCl2, 1 mM DTT and 100 mM NaCl, then fragmented at 94 °C for 30 min with no shaking. Samples were cooled quickly on ice. MNPs and MNs were diluted in protein resuspension buffer, then sonicated for 15 cycles using the BioRuptor plus (Diagenode) and finally heated at 80 °C for 15 min, 300 RPM. Sonication was used in this instance to aid in resuspension of the protein-RNA complexes as well as fragment RNA to the desired size range. Sonication was conducted twice after each phase separation.

#### Phase separation 2

To deplete non-protein-bound RNA after fragmentation, samples were phase separated a second time using QIAzol and chloroform. After separation, the aqueous phase was replaced with nuclease-free water and samples were mixed and re-centrifuged. The aqueous phase was removed again, and the entire interphase and organic phase were precipitated using 9 × ice-cold methanol. This was to ensure that any protein transferred to the organic phase due to the short length of bound RNA, or large protein size, was also recovered. Precipitated protein-RNA complexes were washed and resuspended as before.

#### Exoribonuclease digestion

Please note, this section is demonstrative of a sample size of 2–300,000 HEK293T cells. Reagents should be scaled appropriately to the sample size used.

SDS from the protein resuspension buffer was sequestered using an equal volume of 20% TX-100. Samples were then diluted in 1 × T4 DNA ligase buffer with 10 mM ATP (NEB B0202A), 5 U DNase I (NEB M0303), 10 U T4 PNK (NEB M0201), 5 U XRN1 (NEB M0338) and 60 U murine RNase inhibitor (NEB M0314S). Samples were incubated for 1 h at 37 °C.

#### Protein digestion and RNA isolation

Immediately following exoribonuclease digestion, the protein was digested using 5.3 mg/mL proteinase K (NEB T2001-1) for 2 h, 53 °C, 300 RPM. RNA was isolated using the Monarch RNA cleanup kit (NEB T2040) as per manufacturer instructions.

#### Input preparation

A representative sample of whole cell lysate (*isolated prior to phase separation for MNP/MN samples or reconstituted using protein-bound and ‘free’ RNA from the aqueous phase for HEK293T samples*) was precipitated in 9 × ice-cold methanol, washed and resuspended as described above. Protein was immediately digested, then RNA isolated as described above. The isolated RNA was fragmented and then treated with DNase and PNK, prior to the final enzymatic cleanup.

### Sequencing

DNA libraries were prepared using the NEBNext rRNA depletion kit (NEB, E6310) and NEBNext Small RNA library kit (E7330) according to manufacturer instructions. RNA fragment sizes were assessed by RNA pico chip (Agilent, 5067–1513). Libraries were selected for fragments ranging from 200 to 400 bp equivalent to RNA fragments measuring 80–280 nt. Sequencing was performed at the Exeter sequencing centre using the NovaSeq platform.

#### Peak calling and differential analysis

Reads were mapped to the hg19 genome using STAR [43]. Initial peak calling was performed for each sample using CLIPper [14, 15] and peaks significant at 1e − 8 were retained. Peaks with genomic overlap in at least two ePRINT samples were identified and their genomic coordinates merged to define the candidate peak superset (204,736 peaks) using HOMER [44]. Of these, 192,010 were identified as intragenic when mapped to hg19 gene annotations. These peaks were then filtered using the input, retaining those significantly enriched in the ePRINT samples in comparison to the corresponding input samples (*p*-value < 0.001, 50,342 peaks). Peaks with cumulative read counts < 50 across all four ePRINT samples were removed, retaining 41,980 peaks. Finally, peaks mapping to genes with cumulative counts across all four input samples < 50 (lowly expressed genes) were discarded generating 41,567 peaks. These high confidence peaks were then input to DESEQ2 for differential analysis [45]. The design formula incorporated input base gene expression for each ePRINT peak as a covariate. Differentially bound protein-RNA sites were identified using a statistical model that normalises the change in ePRINT peak amplitude to the change in host gene expression. For this reason, intergenic peaks were not included in the differential peak analysis. For the FUS data, we also included batch as an additional covariate. To generate the expanded peak set for FUS ePRINT, we lowered the CLIPper p-value threshold to 1e − 4 detecting 337,125 peaks. After input filtering at a lower *p*-value threshold of 0.01, we identified 90,784 peaks. Further filtering on peak and gene counts as above retained 89,887 peaks. For the MNP/MN analysis, one of the replicates for the MN input displayed significantly higher percentages of ribosomal reads and was excluded from further analysis. An adjusted *p*-value 0.01 and fold change of 2.0 were used to determine differential peaks. Hypothesis testing and *p*-values were generated using the Wald test with multiple testing corrections using the Benjamini and Hochberg method. Differentially expressed genes were identified using conventional DESeq2 analysis on the input samples.

### Peak visualisation

Bam files were converted into bedgraph tracks using samtools and read density was normalised to sequencing depth for visualisation in the UCSC genome browser [46] as individual bedgraph tracks.

### Mutational analysis

Mutation calling was conducted using GATK RNA sequencing variant calling workflow and known mutations (SNPs or deletions) were identified using the 1000 genomes database [8].

### Motif analysis

For the motif location analysis, peaks were scanned for the presence of RBP motifs in a 200-bp window centred on the peak start (0). Motif position weight matrices (PWM) were derived from the Transite database [36]and motif scanning was performed using the universalmotif R package with a relative log-odds threshold of 0.8 [47]. Scores were averaged for each motif across all peaks at each bp and converted to Z scores for plotting. For motif enrichment analysis, we only scanned a 50-bp window centred on the peak start site.

To identify if any of the RBP motifs were associated with an increase or decrease in binding events, we sorted the peaks from most enhanced to most reduced. The set of peaks identified as carrying a given motif was deemed as a peakset for that particular motif. Enrichment was calculated by running a directional gene set enrichment analysis (GSEA) for each motif using the fgsea package [48].

To calculate motif enrichment in individual gene features, peak start sites and end sites were annotated separately using the CHIPpeakAnno package [49] as mapping to one of the following gene features: exon, intron, 5′ UTR, 3′ UTR. If a peak start and end site lay within an exon, the peak was called exonic. If the peak start mapped to an exon and the end mapped to an intron, the peak was identified as spanning junctions (EX-IN). Similar rules were applied for other features. Peaks matching to RBP motifs were probed for enrichment or depletion of genomic loci (intron, exon, UTR(s) and intron–exon junctions) by hypergeometric test.

To identify de novo sequence motifs in ePRINT and FUS eCLIP peaks, we analysed a 50-bp window centred on ePRINT start sites or FUS eCLIP window centres. To identify structural motifs, we used a 101-bp window. We used PRIESTESS [26] that uses STREME [50] to perform motif enrichment analysis.

### eCLIP and overlap analysis

FUS eCLIP was performed as described by Van Nostrand et al. (2020) [11]. Reads were mapped to human genome hg38 and significant FUS binding sites were identified using Skipper [51]. Sites were then lifted over to hg19 using the UCSC liftOver tool. Sites were considered significant if they had a *p*-value < 0.001 and an enrichment > 2 (4344 enriched windows total). De novo motif analysis was performed using these 4344 enriched windows. For overlap analysis with ePRINT peaks, we only retained FUS eCLIP binding sites that mapped within hg19 annotated genes, resulting in 4121 windows. We identified ePRINT peaks that were within 200 bp of any FUS eCLIP peak. These ePRINT peaks were used as a “geneset” in the GSEA where ePRINT peaks were sorted from most enhanced to reduced. Similar, overlaps were calculated for the ENCODE eCLIP analysis. GSEA was performed using the R fgsea package [48].

### Network analysis

Direct RNA targets of FUS, including downstream RBPs (FUS-RBPs), were identified using FUS CLIP data from HEK293T cells (see eCLIP methods). For Fig. 1N, downstream RNA targets of FUS-RBPs were identified using ENCODE eCLIP datasets, which were obtained from www.encodeproject.org. Downstream targets of FUS-RBPs in Fig. 1O were identified using ePRINT motif analysis (Transite). In Fig. S7, all RBP targets were identified using the RBNS database motifs. Networks were plotted in R using the GGally extension [52] to the ggplot2 package [53]. Simulations were completed by generating 53 random genes from the HEK293T transcriptome, to compare to the 53 differentially expressed genes after FUS knockdown.

### Sequence clustering and kmer analysis

ePRINT peaks were first separated into enhanced or reduced peaks. Pairwise alignments were conducted between these peak sequences and all potential 6mers using the Biostrings package [54]. Peaks within the reduced and enhanced sets were clustered separately based on alignment scores using hierarchical clustering, such that peaks containing similar 6mers were grouped together. Hypergeometric tests were deployed to identify kmers enriched within clusters, and then these kmers were checked against the Transite database [36] to determine if they were attributed to particular RBPs. Any unknown kmers were re-assessed by GSEA to confirm enrichment in up/down peaks.

### iPSC maintenance and MN differentiation

Healthy human iPSCs (GM23279A) were obtained from the Coriell Institute for Medical Research. iPSCs were maintained as colonies on human ES-qualified Matrigel (Corning) in StemFlex (StemCell Technologies). Colonies were routinely passaged using EDTA and mycoplasma testing was conducted regularly to rule out contamination of cultures.

For differentiation to MN, iPSCs were plated as colonies onto Matrigel and treated with neuronal differentiation media (DMEM/F12:Neurobasal in a 1:1 ratio, HEPES 10 mM, N2 supplement 1%, B27 supplement 1%, L-glutamine 1%, ascorbic acid 5 μM) supplemented with SB431542 (40 μM), CHIR9921 (3 μM) and LDN8312 (0.2 μM) from day 0 till day 4. Cells were caudalized by treatment with 0.1 μM retinoic acid starting on day 2 and ventralized with 1 μM purmorphamine starting on day 4 and continued till day 8. On day 8, motor neuron progenitors (MNPs) were re-plated onto poly-D-lysine/laminin-coated wells. Differentiation was induced by treating the cells with N2B27 media supplemented with retinoic acid, purmorphamine and DAPT 10 μM. DAPT treatment was stopped at day 13 and media was changed to N2B27 supplemented with BDNF 10 μg/ml and GDNF 10 μg/ml. Samples were collected on day 10 (MNP) and day 15 (MN) for ePRINT.

### RBP overexpression

HNRNPF, SRSF9 and eGFP mRNA sequences were inserted into the inducible pLV-TetO vector, replacing NGN2. Viral particles were produced using the pspax2 packaging vector and the pMD2.G envelope vector. The three expression vectors (eGFP, SRSF9 and HNRNPF) were transfected into HEK293T cells along with the packaging plasmids at equal amounts to generate the lentiviruses. The tet-encoding virus pLV_hEF1a_rtTA3 was generated in parallel. We added 0.625 μL of each virus to 10,000 progenitors in a 96-well that had 50 μL of culture media. These conditions were kept the same for all three constructs. MNPs were transduced at day 9 and RBP expression was induced with 2 μg/mL doxycycline (dox) at day 10 (Fig. S13). Notch inhibition was conducted at day 11 using DAPT as described above and maintained until day 16 when cells were fixed for immunofluorescence. Twenty-four hours prior to fixation, cells were selected with 500 ng/mL puromycin. Dox-containing media was replenished every 48 h.

p3x-FLAG-hnRNPF was a gift from Mariano Garcia-Blanco, addgene #21,926 [55]. SRSF9_2xRRM_pGEX was a gift from Christopher Burge, addgene #135,120 [56]. pLV-TetO-hNGN2-Puro was a gift from Kristen Brennand addgene #79,049 [57]. psPAX2 and pMD2.G were gifts from Didier Trono (Addgene plasmid #12,260 and #12,259). pLV_hEF1a_rtTA3 was a gift from Ron Weiss addgene #61,472 [58].

### Gene ontology

Leading edge peaks matching the HNRNPF or SRSF9 associated motif(s) were mapped to genes to form our gene sets of interest (see the ‘ Motif analysis’ section). Using these gene sets, against all genes with an identifiable peak, we probed for enrichment of biological processes using the ClusterProfiler package in R [59].

### Supplementary Information

Additional file 1: Supplementary figures: Fig. S1 – Fig. S15 and Tables S1 and S2.

Additional file 2. Supplementary data file as a single excel worksheet with four tabs labelled: eprint vs FUS gene targets, FUS siRNA vs ctrl siRNA DEG, Unknown kmer enrich FUSeprint, MN vs MNP RBP DEG.

Additional file 3. Review history.

## Data Availability

The ePRINT (GSE230097) and eCLIP (GSE266924) datasets have been deposited to the Gene Expression Omnibus database [60].
