# Supplementary material for: ePRINT: exonuclease assisted mapping of protein-RNA interactions
Source: Genome Biol. 2024 May 28;25:140. doi: 10.1186/s13059-024-03271-1 (PMC11134894; doi:10.1186/s13059-024-03271-1)
Supplement: Supplementary file 1 — Additional file 1: Supplementary figures: Fig. S1 – Fig. S15 and Tables S1 and S2. [file 13059_2024_3271_MOESM1_ESM.pdf]

Fig S1

A

UV mJ/cm2	µg RNA in aqueous phase	µg RNA in interphase	RNA in aqueous phase	RNA in interphase
0	23.76	2.8	89.5 %	10.5 %
200	3.37	12.76	20.9 %	79.1 %
400	1.16	10.93	9.6 %	90.4 %

B

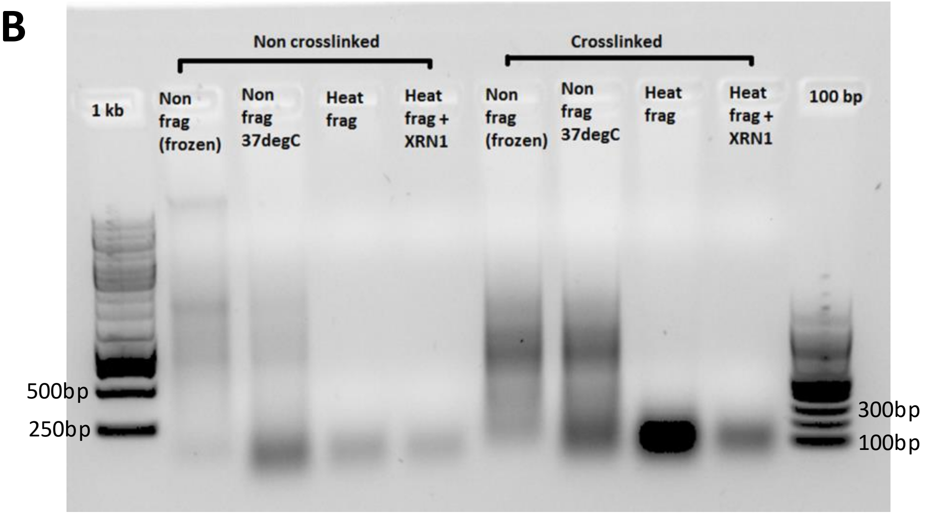

Fig S1. ePRINT experimental optimizations

A) RNA is depleted from the aqueous phase upon UV irradiation and can be recovered from the interphase. In brief: HEK293T cells were irradiated at 0, 200 and 400 mJ/cm^2, then lysed and phase separated using QIAzol and chloroform. For the aqueous phase, free RNA was extracted using spin column purification. For the isolated interphase, protein was digested using proteinase K, then the RNA was isolated using spin column purification with gDNA removal. Both the aqueous and interphase extractions included DNase treatments.

B) RNA abundance isolated from interphase is increased upon UV irradiation (400 mJ/cm^2). Heat fragmentation for 30min, 94degC is optimal to reduce RNA size to ~100-200bp. XRN1 greatly reduces RNA abundance in crosslinked cells, indicating successful 5'-3' digestion.

Fig S2

Examples of peaks removed during input filtering.

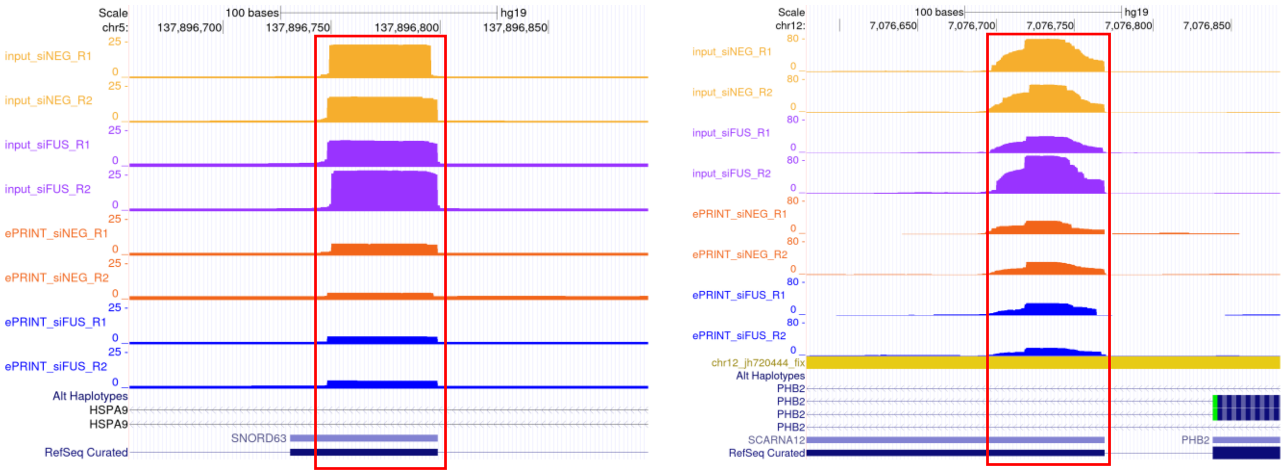

Examples of peak retained during input filtering.

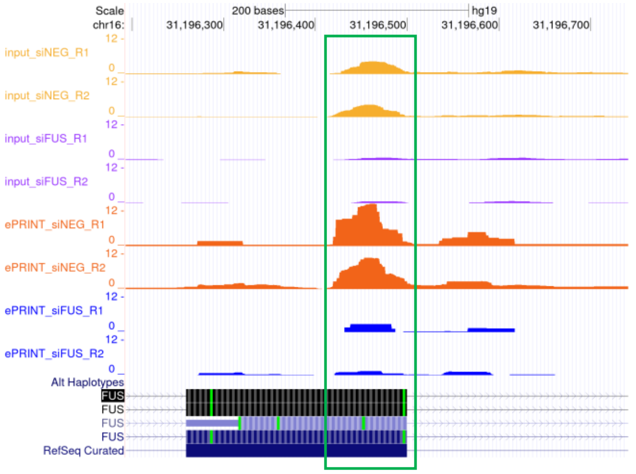

Fig S2. Representative UCSC browser screenshot showing the importance of input filtering.

Top panel: Examples of peaks removed during input filtering, i.e. we remove peaks that are not enriched in ePRINT samples, and therefore unlikely to represent RBP binding events.

Bottom panel: Example peak that is retained during input filtering. Peaks must be enriched in ePRINT samples vs input samples, but may still be present in input. During differential peak analysis, the input is used to normalise any change in peak amplitude to the base gene expression.

Fig S3

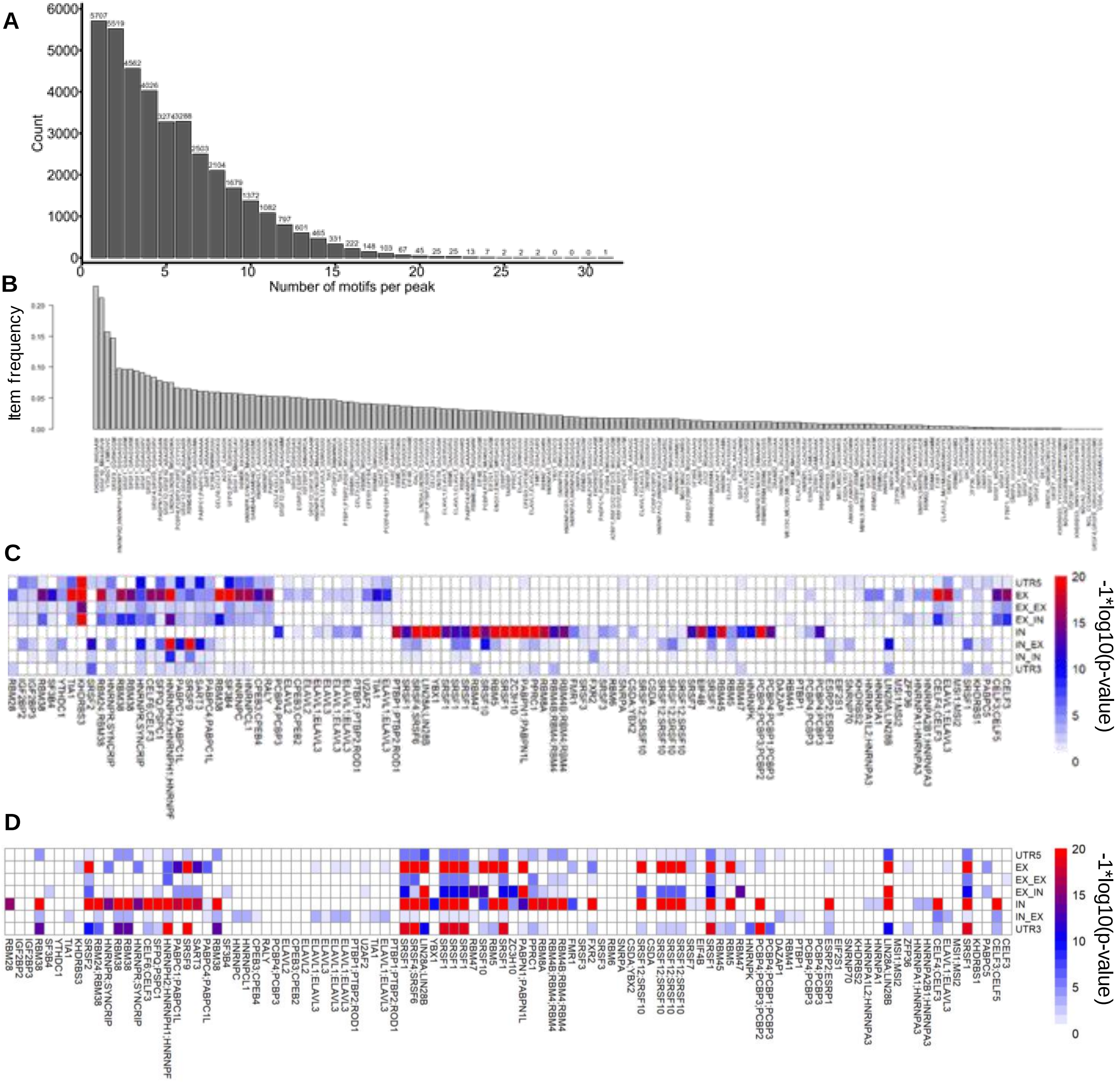

Fig S3. ePRINT binding site distribution across gene features

A) Number of motifs associated with each ePRINT peak.

B) Frequency of motifs detected per ePRINT peak. X axis indicates RBP motifs, Y axis indicates the proportion of peaks. Motifs are ordered from most to least promiscuous.

C) RBP motifs identified as depleted in specific gene features by hypergeometric test. Associated with Fig.1H.

D) RBP motifs identified as enriched in specific gene features based on randomly selected peaks within each gene feature. Associated with Fig.1H.

Fig S4

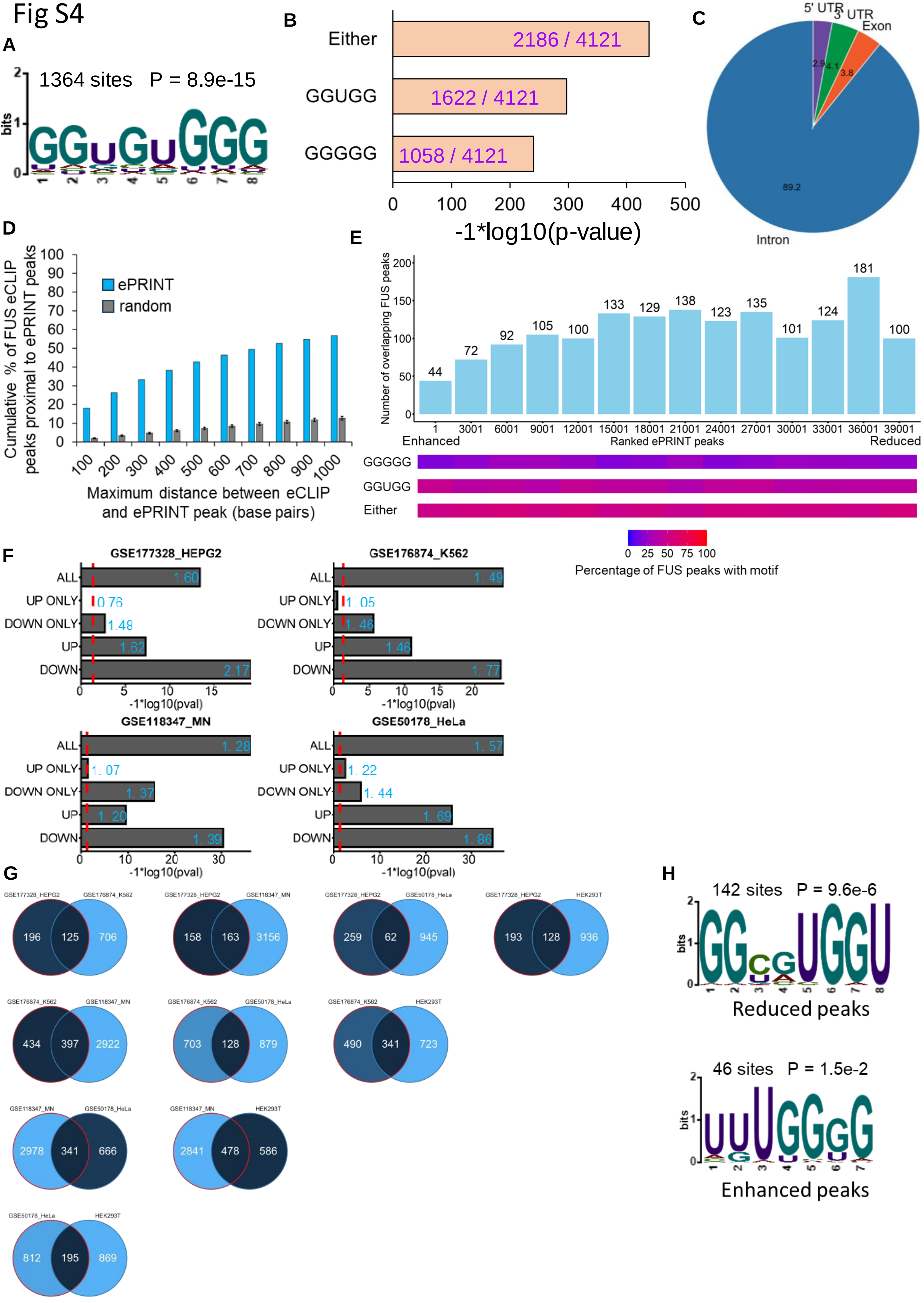

A
1364 sites P = 8.9e-15
bits
B
Either
GGUGG
GGGGG
2186 / 4121
1622 / 4121
1058 / 4121
0 100 200 300 400 500
-1*log10(p-value)
C
5' UTR
3' UTR
Exon
Intron
2.9
4.1
3.8
89.2
D
Cumulative % of FUS eCLIP peaks proximal to ePRINT peaks
ePRINT
random
Maximum distance between eCLIP and ePRINT peak (base pairs)
E
Number of overlapping FUS peaks
44 72 92 105 100 133 129 138 123 135 101 124 181 100
1 3001 6001 9001 12001 15001 18001 21001 24001 27001 30001 33001 36001 39001
Enhanced
Ranked ePRINT peaks
Reduced
GGGGG
GGUGG
Either
0 25 50 75 100
Percentage of FUS peaks with motif
F
GSE177328_HEPG2
GSE176874_K562
GSE118347_MN
GSE50178_HeLa
ALL
UP ONLY
DOWN ONLY
UP
DOWN
-1*log10(pval)
G
GSE177328_HEPG2
GSE176874_K562
GSE118347_MN
GSE50178_HeLa
HEK293T
H
142 sites P = 9.6e-6
Reduced peaks
46 sites P = 1.5e-2
Enhanced peaks

Fig S4. Overlap analysis of peaks and genes identified using ePRINT and FUS eCLIP
A) Top scoring motif identified by de novo motif enrichment analysis on FUS eCLIP peaks.
B) Enrichment of RBNS FUS motifs identified in FUS eCLIP of HEK293T cells.
C) Distribution of FUS eCLIP peaks identified in HEK293T cells across the following gene features: Introns, Exons, 5' UTRs, 3'UTRs. Numbers indicate percentages of ePRINT peaks mapped to a given feature.
D) Overlap analysis indicating the percentage of FUS CLIP peaks (HEK293T) that are captured by ePRINT peaks vs randomly selected peaks within the transcriptome. X axis indicates the maximum distance between the centre of a CLIP peak and ePRINT peak start site. Y axis show the percentage of FUS eCLIP peaks overlapping with ePRINT. At a distance of 200 bp, ePRINT captures ~25% of FUS eCLIP peaks.
E) Overlap analysis to determine the abundance of FUS eCLIP peaks that overlap with enhanced, unchanged or reduced ePRINT peaks. X axis indicates ePRINT peaks ranked and binned into groups from most enhanced to most reduced. Y axis indicated the number of FUS eCLIP peaks found within each bin of ePRINT peaks. X axis heatmap indicates the percentage of eCLIP peaks within each bin that contain a canonical FUS motif.
F) Overlap analysis to determine enrichment of FUS targets in genes that display enhanced (UP) or reduced (DOWN) ePRINT peaks upon FUS knockdown. Genes carrying peaks in both directions are excluded in the UP ONLY and DOWN ONLY comparisons. Datasets and the corresponding cell lines have been indicated at the top of each bar plot. Values shown in blue indicate fold enrichment of FUS targets in each ePRINT peak group vs background. Associated with Fig.1L.
G) Venn diagrams showing the pairwise analysis of the overlap between FUS target genes between CLIP datasets.
H) Top scoring motif identified by de novo motif enrichment analysis on the reduced (top panel) and enhanced (bottom panel) ePRINT peak sets.

Fig S5

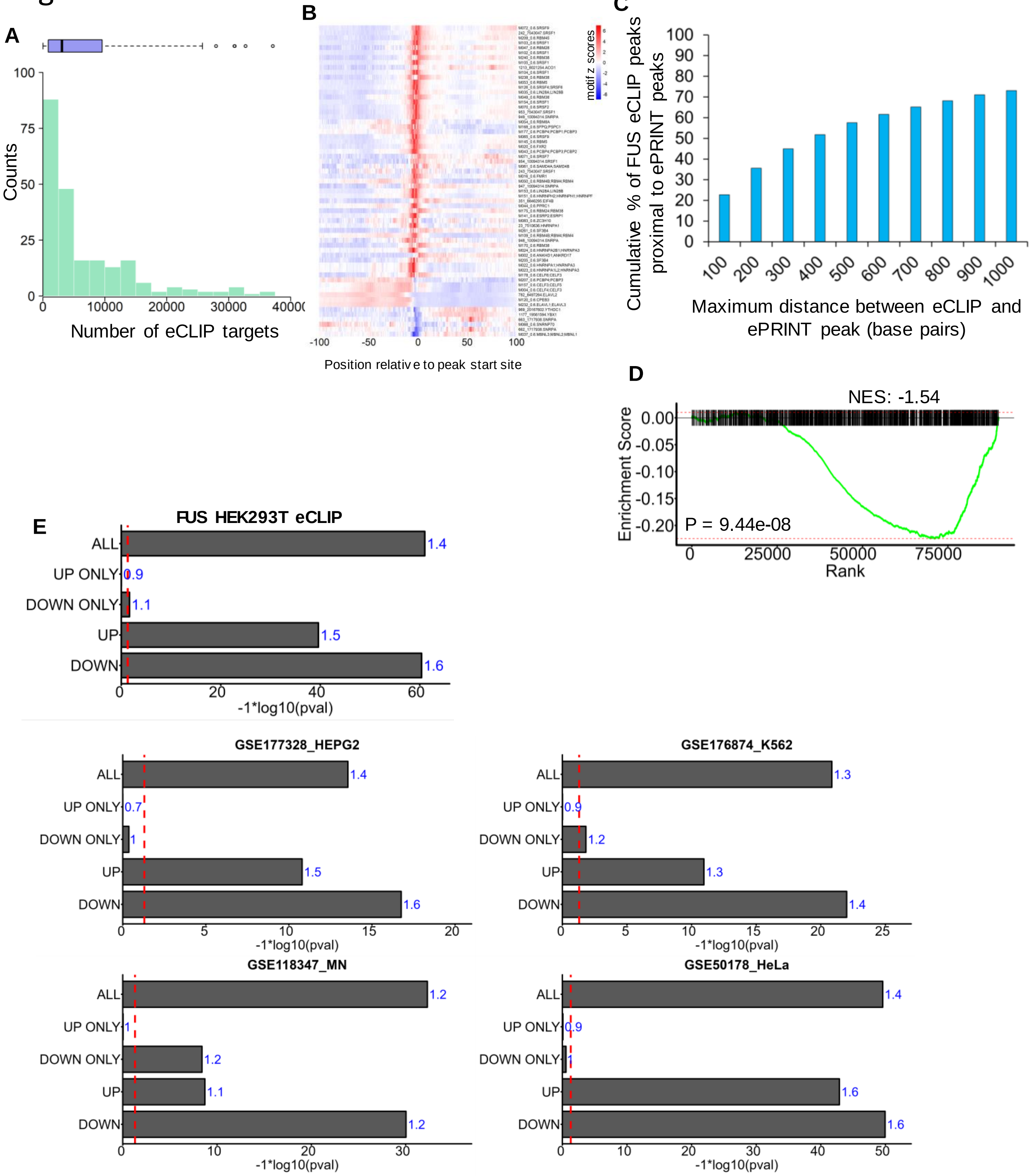
A
100
75
50
25
0
Counts
0
10000
20000
30000
40000
Number of eCLIP targets
B
motif z scores
-100
-50
0
50
100
Position relative to peak start site
C
Cumulative % of FUS eCLIP peaks proximal to ePRINT peaks
100
90
80
70
60
50
40
30
20
10
0
100
200
300
400
500
600
700
800
900
1000
Maximum distance between eCLIP and ePRINT peak (base pairs)
D
NES: -1.54
Enrichment Score
0.00
-0.05
-0.10
-0.15
-0.20
P = 9.44e-08
0
25000
50000
75000
Rank
E
FUS HEK293T eCLIP
ALL
UP ONLY
DOWN ONLY
UP
DOWN
1.4
0.9
1.1
1.5
1.6
0
20
40
60
-1*log10(pval)
GSE177328_HEPG2
ALL
UP ONLY
DOWN ONLY
UP
DOWN
1.4
0.7
1
1.5
1.6
0
5
10
15
20
-1*log10(pval)
GSE176874_K562
ALL
UP ONLY
DOWN ONLY
UP
DOWN
1.3
0.9
1.2
1.3
1.4
0
5
10
15
20
25
-1*log10(pval)
GSE118347_MN
ALL
UP ONLY
DOWN ONLY
UP
DOWN
1.2
1
1.2
1.1
1.2
0
10
20
30
-1*log10(pval)
GSE50178_HeLa
ALL
UP ONLY
DOWN ONLY
UP
DOWN
1.4
0.9
1
1.6
1.6
0
10
20
30
40
50
-1*log10(pval)

Fig S5. Expanded peak set figures

A) Distribution of the number of targets for various RBPs identified using eCLIP in the ENCODE data.

B) RBP motifs are enriched at peak start sites indicated by 0 on the x-axis. Peak start sites were extended by 100bp (+/-). Scores for individual motifs were estimated at each bp along each peak using the position weight matrices. Per bp scores were averaged across all peaks and converted into z-scores: higher z-scores (red) indicate higher probability of locating the motif(s). Left panel indicates peaks identified in ePRINT samples

C) Overlap analysis indicating the percentage of FUS CLIP peaks (HEK293T) that are captured by the 89,897 ePRINT peaks. X axis indicates the maximum distance between the centre of a CLIP peak and ePRINT peak start site. Y axis show the percentage of FUS eCLIP peaks overlapping with ePRINT.

D) Peak set enrichment analysis of FUS binding sites identified using eCLIP in HEK293T cells. X-axis indicates ePRINT peaks ranked from most significantly upregulated (left side) to most significantly downregulated (right side).

E) Hypergeometric test to determine enrichment of FUS target genes identified using eCLIP in different cell lines in genes that display enhanced (UP) or reduced (DOWN) ePRINT peaks upon FUS knockdown. Genes with peaks in both directions are excluded in the UP ONLY and DOWN ONLY comparisons. Values shown in blue indicate fold enrichment of FUS targets in each ePRINT peak group vs background. FUS eCLIP datasets used are displayed on the top of each bar chart.

Fig S6

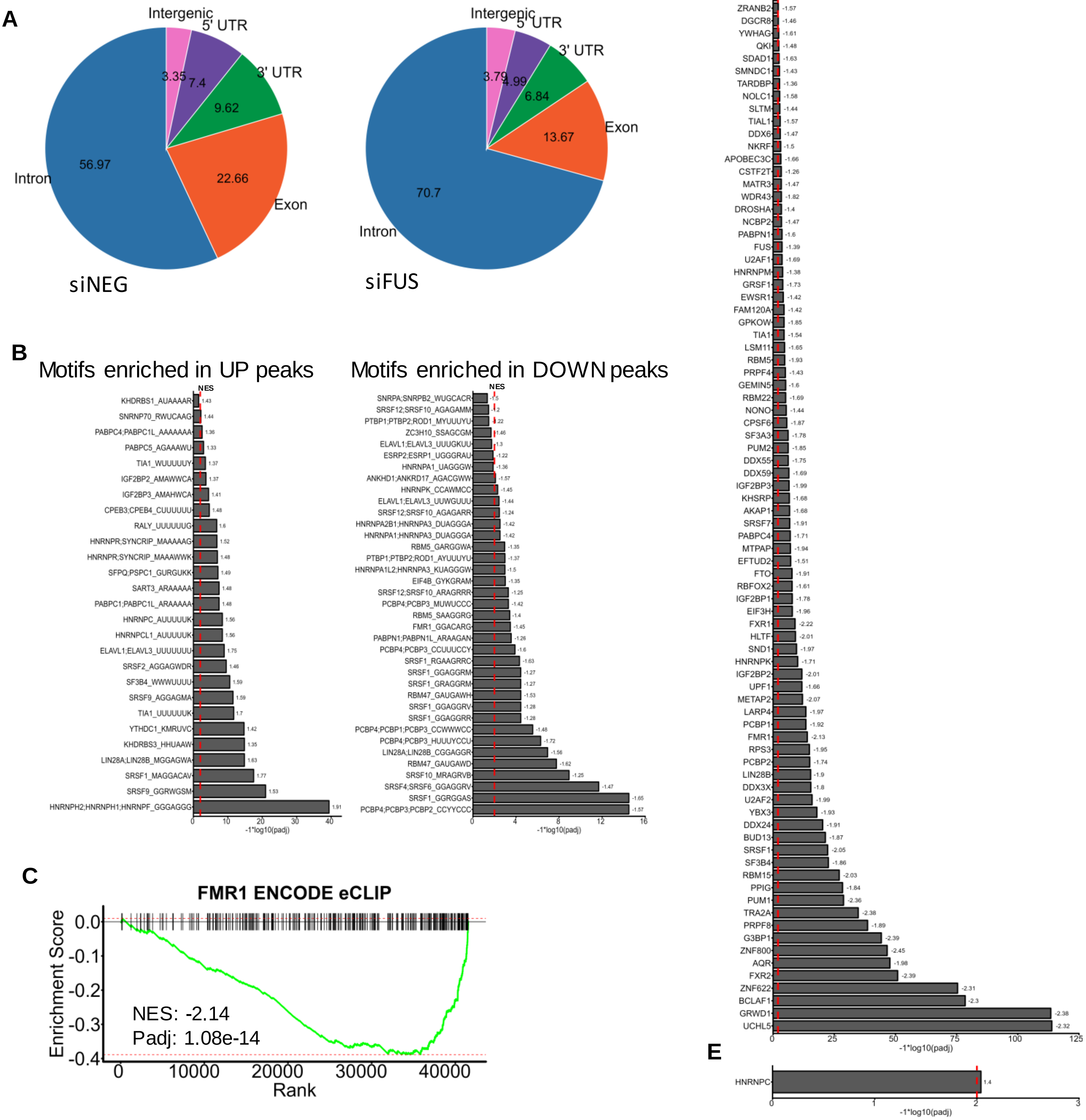

Fig S6. Motif analysis of ePRINT peaks

A) Distribution of ePRINT peaks mapped to gene features. Numbers indicate percentages of ePRINT peaks mapped to a given feature. Pie charts display data for siNEG and siFUS conditions.

B) All RBP motifs identified as enriched in peaks that are enhanced (UP) or reduced (DOWN) in amplitude after FUS knockdown (adjusted p-value threshold of 0.05). Red dotted line indicates a p-value threshold of 0.01. Associated with Fig.1M. Normalised enrichment score (NES) estimated by GSEA is included for each comparison.

C) Peak set enrichment analysis of FMR1 binding sites identified using eCLIP in K562 cell line. FMR1 eCLIP data was obtained from ENCODE. X-axis indicates ePRINT peaks ranked from most significantly upregulated (left side) to most significantly downregulated (right side). Size indicates the number of ePRINT peaks overlapping with FMR1 peaks.

D, E) RBPs identified as decreased (D) or increased (E) in activity by GSEA analysis, using encode eCLIP genesets against ePRINT peaks after FUS depletion. NES indicates normalised enrichment score estimated by GSEA.

Fig S7

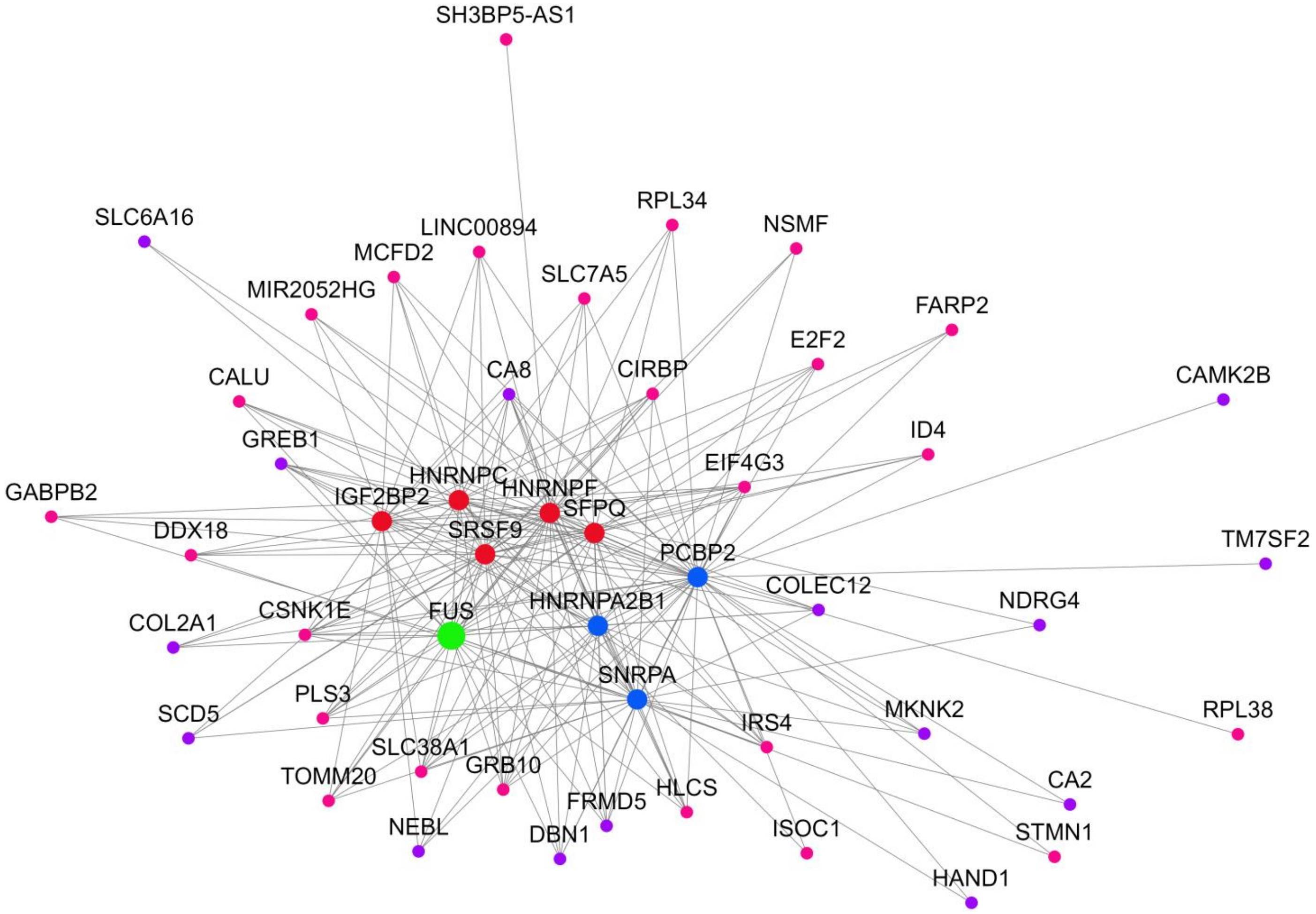

Number of differentially expressed genes captured	41
Number of network edges	244

- FUS
- RBP with increase in binding events
- RBP with decrease in binding events
- RBP with increase and decrease in binding events
- Upregulated gene
- Downregulated gene

Fig S7. Network analysis to identify direct and indirect effects of FUS knockdown using RBNS and ePRINT data. Motifs (5-mers) included in the RBNS database were used to identify direct targets of FUS within ePRINT peaks, including differentially expressed genes (DEGs) and RBPs with a change in activity shown in figures 1M and S6B (FUS-RBPs). RBNS data was then used to identify links from FUS-RBPs to DEGs.

Colour legend indicates expression changes for RBPs and genes.

Fig S8

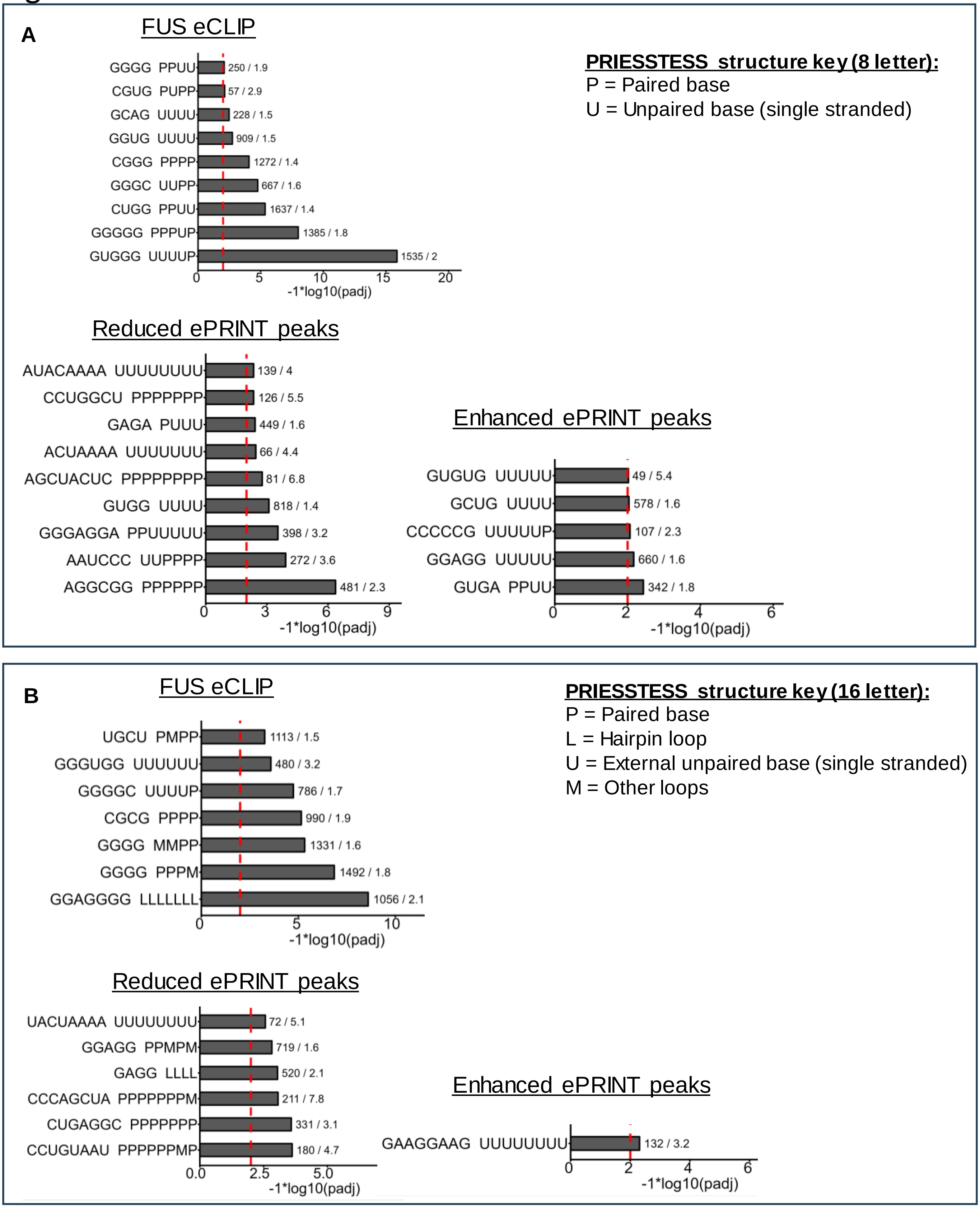

Fig S8. Sequence and structure motif enrichment analysis

A, B) Sequence-structure motifs enriched in FUS eCLIP (top), reduced ePRINT peaks after FUS knockdown (bottom left) and enhanced ePRINT peaks after FUS knockdown (bottom right).
A) Enrichment was determined using the 8 letter alphabet from PRIESSTESS (see key). B) Enrichment was determined using the 16 letter alphabet from PRIESSTESS (see key).

Numbers to the right of each bar indicates: number of sites with this motif / fold enrichment over background
Red line indicates padj value of 0.01.

Fig S9

A

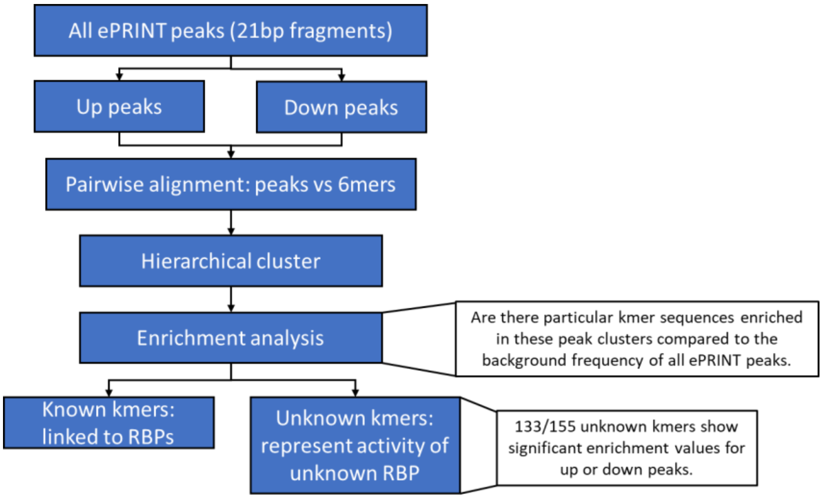

B

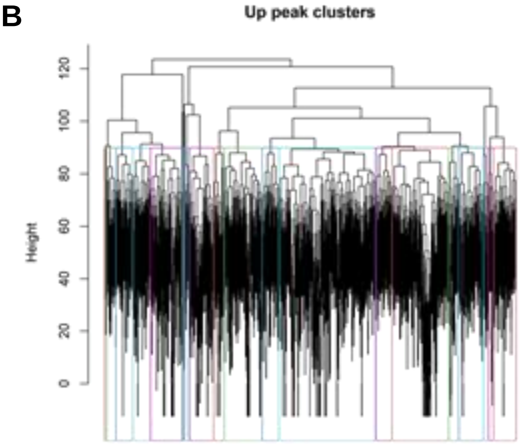

C

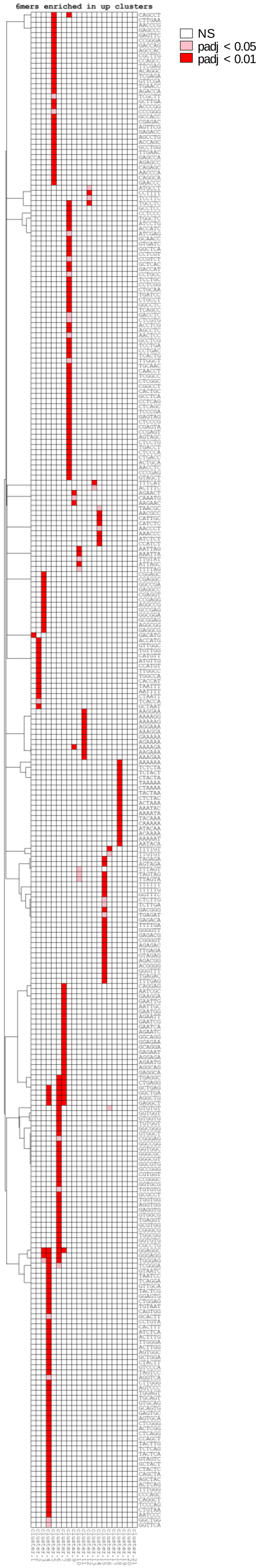

D

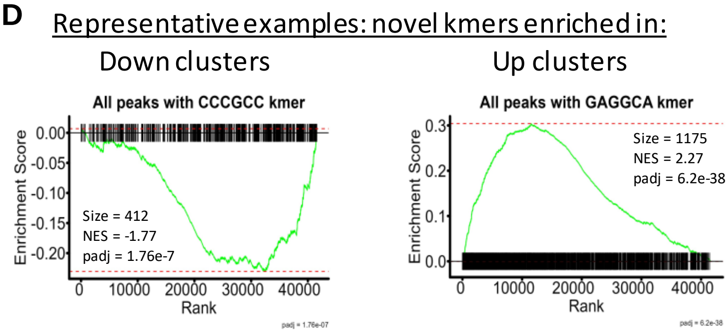

Fig S9. Clustering analysis to identify new motifs

A) Flow chart depicting analytical approach

B) Cluster dendrogram of all ePRINT peaks identified to have an increase in amplitude after FUS knockdown. Clusters are indicated by coloured rectangles.

C) Hypergeometric tests indicating enrichment of kmers within up peak clusters. Padj indicates Benjamini and Hochberg multiple corrections test.

D) Example GSEA plots confirming enrichment of unknown kmers in peak that are increased or reduced in amplitude after FUS depletion. Size indicates the number of ePRINT peaks that contain the 6-mer.

Fig S10

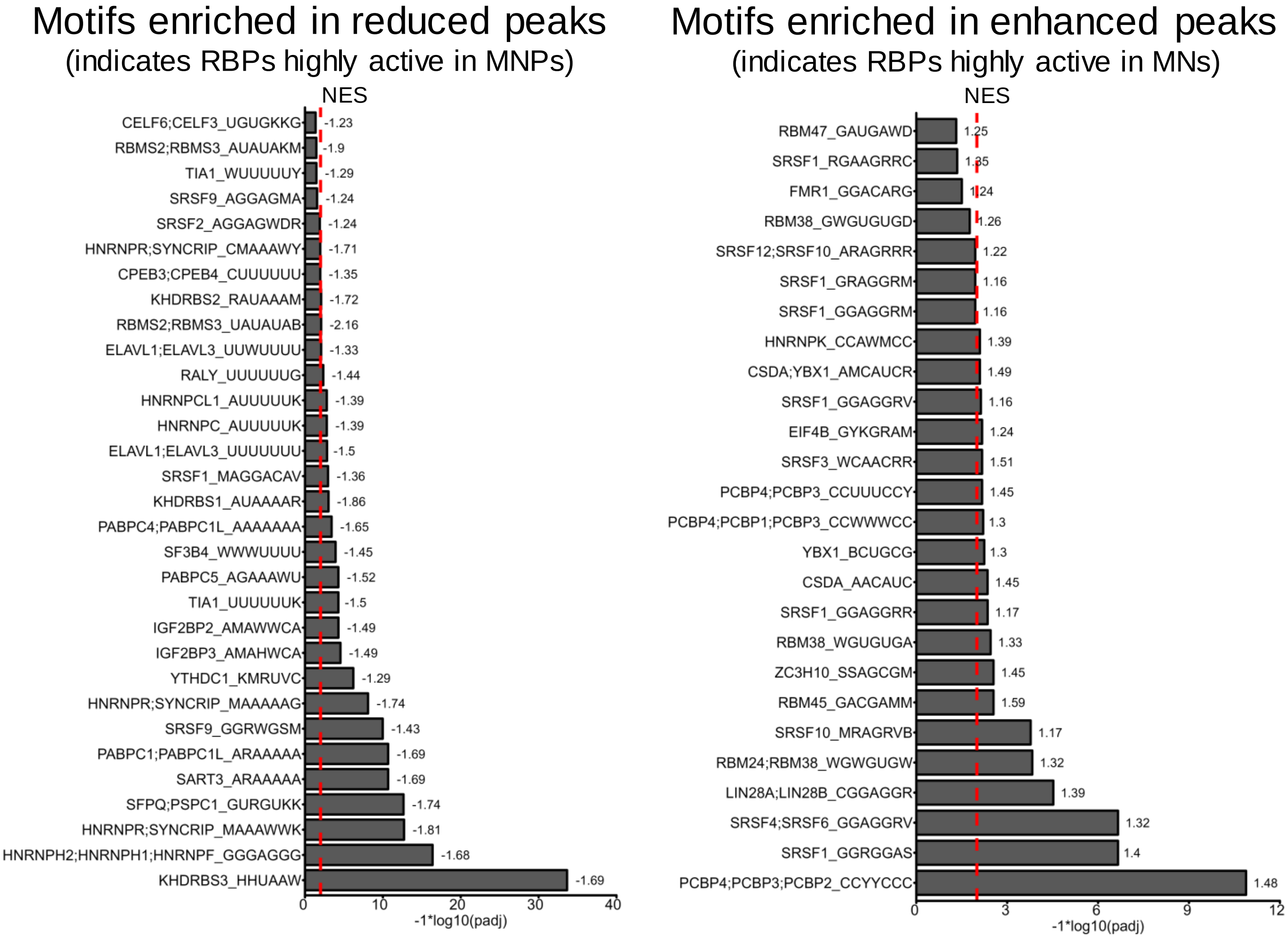

Fig S10. All RBP motifs identified as enriched in peaks that are enhanced or reduced in amplitude in MNs compared to MNPs (adjusted p-value threshold of 0.05). Red dotted line indicates a p-value threshold of 0.01. Associated with Fig.2D. NES indicates normalised enrichment score identified by GSEA.

Fig S11

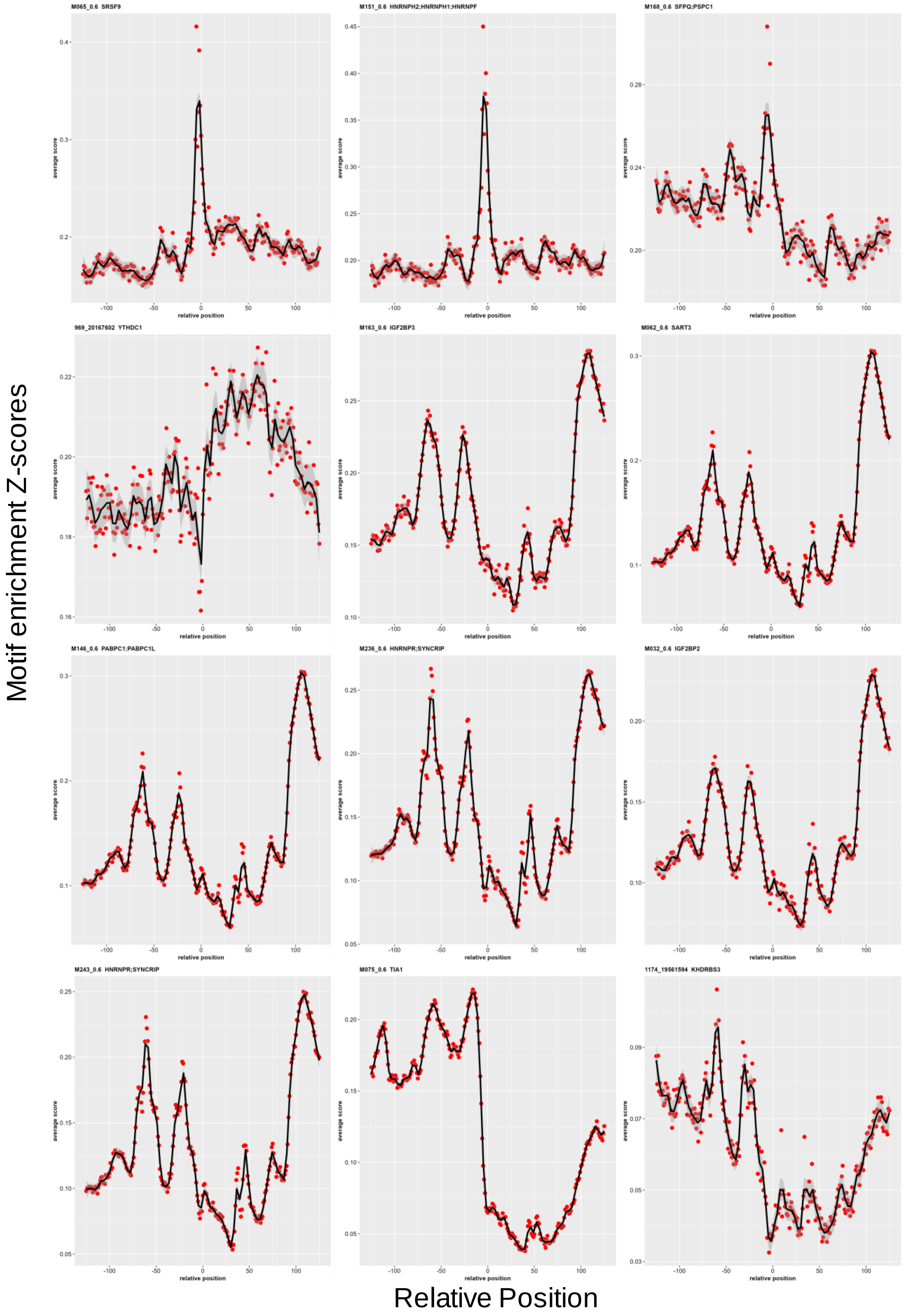

Fig S11. Motif location analysis to determine most probable binding location of RBPs with associated peaks reduced in MNs. A sharp peak at 0 indicated enrichment of binding location at peak start site. Graphs are ordered by enrichment score at 0 (high to low).

Fig S12

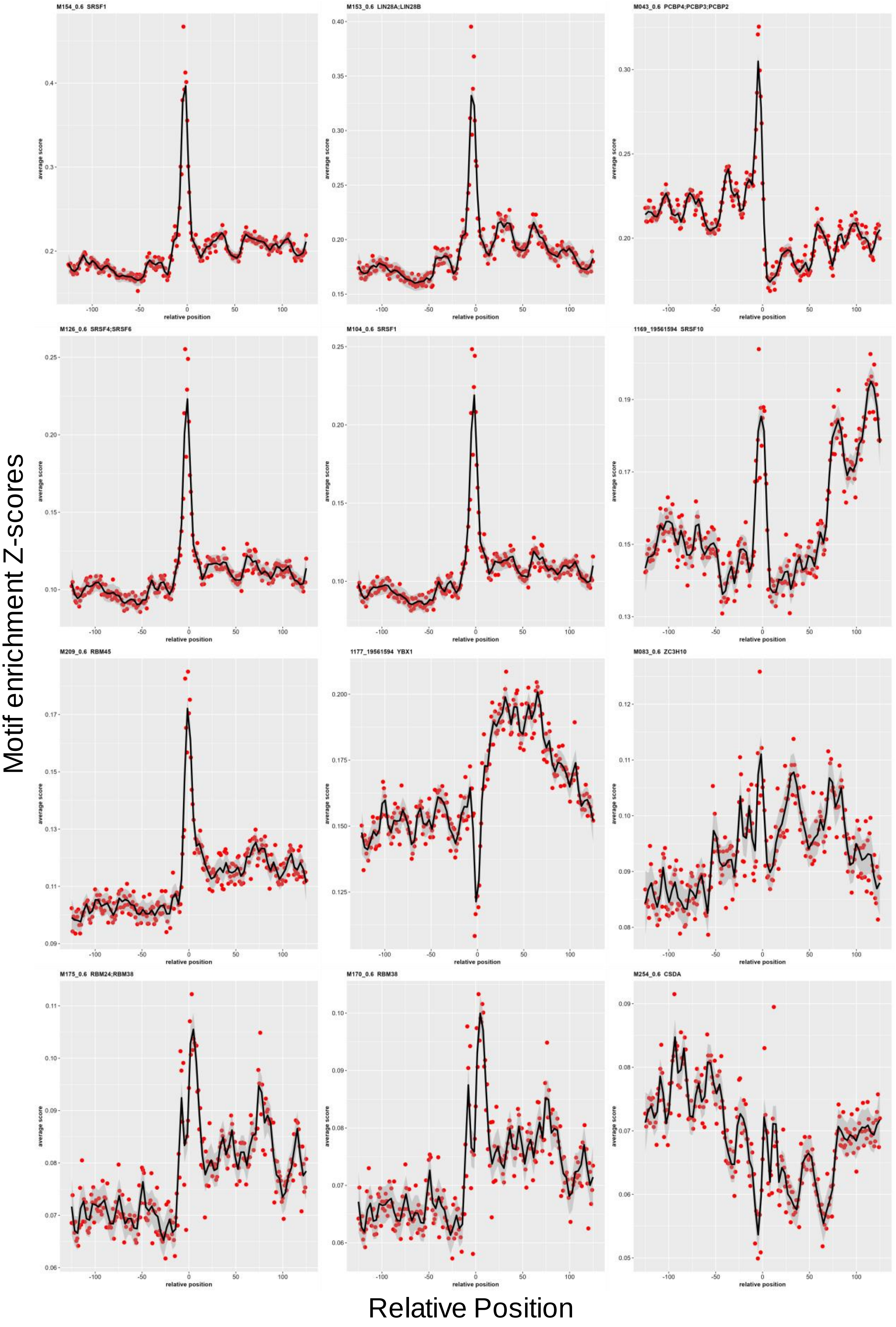

Fig S12. Motif location analysis to determine most probable binding location of RBPs with associated peaks enhanced in MNs. A sharp peak at 0 indicated enrichment of binding location at peak start site. Graphs are ordered by enrichment score at 0 (high to low).

Fig S13

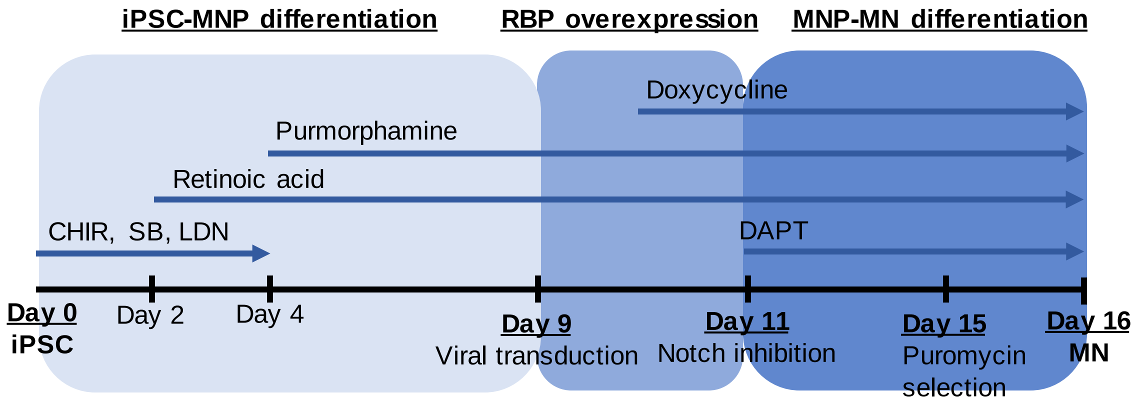

Fig S13. Experimental timeline for RBP overexpression during motor neuron differentiation. RBPs were expressed using dox-inducible viral vectors.

Fig S14

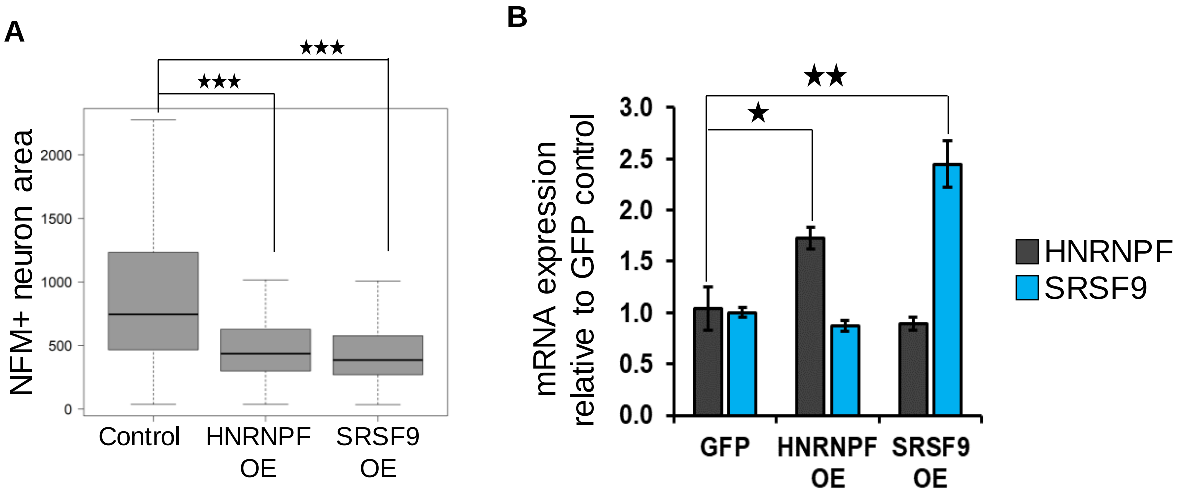

Fig S14. Overexpression of HNRNPF and SRSF9 in motor neuron progenitors inhibits neurite outgrowth. Related to Fig.2F

A) Quantification of neuron size, measured by the area staining positive for NFM for each cell. N = 276 (GFP), 210 (HNRNPF) and 143 (SRSF9).

B) RT-qPCR validation of the overexpression constructs in HEK293T cells. Cells were transduced with dox-inducible and rtTA expressing lentiviruses and incubated for 48 hours in doxycycline-containing media after transduction. Transduction efficiency was ~50%. No antibiotic selection was performed before harvesting cells for RNA. N = 3, error bars indicate SEM.

* indicates pval <0.05. ** indicates pval <0.01. *** indicates pval <0.001 by Student's t-test.

Fig S15

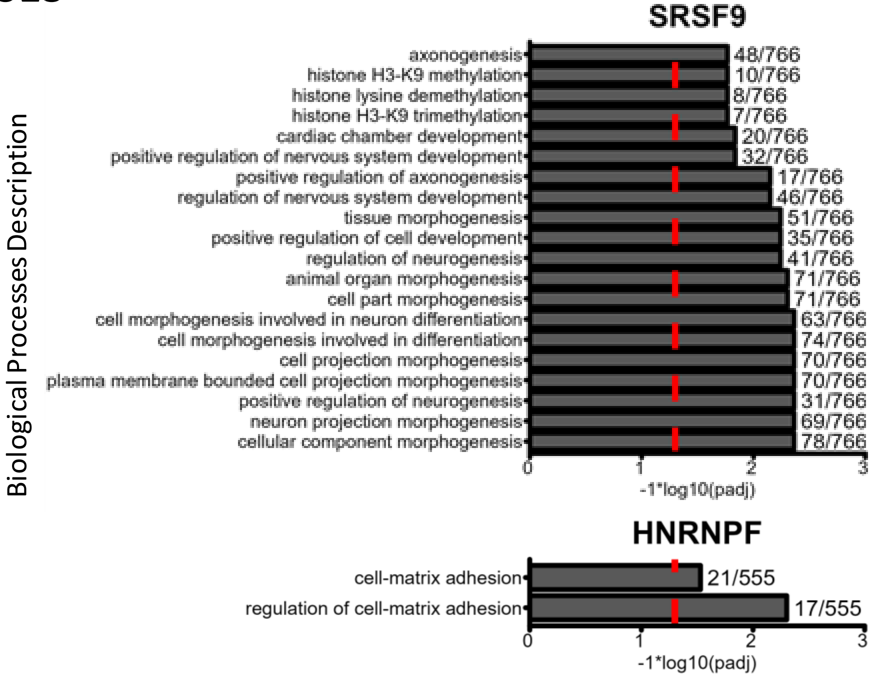

Fig S15. Gene ontology analysis of HNRNPF and SRSF9 target genes. Number of genes linked to a HNRNPF binding site = 623. Number of genes linked to an SRSF9 binding site = 857. Numbers on right hand side (n / N): N indicates the number of genes for that RBP that are also found in the gene ontology database. n indicates the number of genes for that RBP that are linked to the named biological process.

Red line indicates an adjusted p-value of 0.05. Top 20 results shown for SRSF9 (total 34 enriched processes).

	FUS R1	FUS R2	Input R1	Input R2
Total number of sequenced reads	49104396	44385445	36888894	36459532
Uniquely mapped reads	11893564	9681629	6928041	6084572
Uniquely mapped reads %	24.22	21.81	18.78	16.69

Table S1: Sequencing run statistics for the FUS eCLIP samples. R1 and R2 indicate independent replicates.

A

Measurement	FUS (x)	mean (Simulations)	sd (Simulations)	P (X>=x)
No. differentially expressed genes captured	30	28.80	3.68	0.426
No. network edges	100	90.61	10.69	0.191

B

Measurement	FUS (x)	mean (Simulations)	sd (Simulations)	P(X>=x)
No. differentially expressed genes captured	38	31.29	3.57	0.039
No. network edges	336	282.56	24.47	0.019

Table S2: Comparison of the number of differentially expressed genes and network edges captures using eCLIP (A) and ePRINT (B) data.. x indicates values obtained using the differentially expressed genes due to FUS knockdown. Simulations (1000) sample random genes equal to the number of differentially expressed genes. P(X>=x) indicates the probability of observing a value equal or more than x from the random simulations.
