# Supplementary material for: ePRINT: exonuclease assisted mapping of protein-RNA interactions
Source: Genome Biol. 2024 May 28;25:140. doi: 10.1186/s13059-024-03271-1 (PMC11134894; doi:10.1186/s13059-024-03271-1)
Supplement: Supplementary file 3 — Additional file 3. Review history. [file 13059_2024_3271_MOESM3_ESM.docx]

**1^st^ round**

**Reviewer 1**

This study by Hawkins et al reports the development of a method, that they named ePRINT, allowing the global mapping of proteins occupancy on the transcriptome. Using UV crosslinking, organic extraction of RNA in complex with RNA-binding proteins (RBPs), and exoribonuclease treatment on fragmented RNAs, ePRINT is designed to sequence small RNA fragments originally bound by all RBP in vivo. The authors used ePRINT in HEK293T cells to map the different RBP coverage on the transcriptome with or without knockdown of FUS, an intensely studied nuclear RBP. They identified more than 40,000 peaks representing potential binding sites of all RBP in cells. A significant overlap was found between previous FUS CLIP datasets and ePRINT peaks containing FUS RNA binding motifs, suggesting that ePRINT capture true binding events. Using these data, they searched for the presence of hundreds of motifs, known to be preferentially bound by other RBPs, to identify all potential RBP with altered RNA binding following FUS knockdown. They finally applied ePRINT during the cellular state transition of motor neurons differentiation to highlight key RBPs that could be affected in this biological context.

To develop the ePRINT method, the authors combined: (1) the recent observation that UV crosslinked RNA-RBP complexes migrate to the interphase during organic extraction, and (2) XRN1 exoribonuclease treatment of recovered RBP-RNA fragments to restrain the sequencing on the RBP footprint. In this view, the ePRINT workflow is well designed and the proper negative controls are used to remove false-positive signal. However, what contrasts their methods to existing, very similar approaches (e.g. Munschauer et al., Methods 2014 and related Schueler et al., Genome Biol., 2014) is difficult to see or at least is not enough emphasized in the manuscript. One thing that sets the ePRINT apart from previous methods is the use of XRN1 to obtain the footprint, but it is not clear whether this is advantageous as XRN1 could leave a longer 3' end on the RNA fragment compared to classical RNAses I, T1 or A. Furthermore, the authors claim that one advantage of the ePRINT is the absence of individual purification of proteins in the workflow compared to standard CLIP. That is true but at the same time a considerable amount of difficult analysis downstream of ePRINT, using known RNA binding motifs, requires the use of previous datasets established through single purification of proteins. In other words, the full analysis of ePRINT output seem highly dependent on the update of current database and on the presence of clean and interpretable CLIP datasets.

Major points
- The ePRINT output analysis presented by the authors is based on the presence of previously identified RNA-binding proteins motifs in the final obtained peaks. The conclusions that finding such motifs in ePRINT peaks relate a binding site for the associated RBP is a bit simplistic and has the main disadvantage to rely on the quality of previously established data. Hence, this analysis appears limited and biased for already well known RBPs, excluding the analysis of: (1) poorly characterized RBPs; (2) RBPs with preference for specific structures rather than sequences; (3) RBPs competing for the same motif; (4) RBPs with regulated RNA binding preferences. Applying ePRINT seems a promising tool to globally map the fraction of RBP-bound RNAs in different biological contexts but seems difficult to use for measuring the direct impact of individual RBPs if it is only based on the presence of motifs inside peaks. A key question that the author could discuss is: is finding an enriched motif in condition A versus condition B using ePRINT necessarily means an increased binding of the associated RBP? Also, to properly perform differential analysis, ePRINT needs to be exhaustive - how many peaks per transcriptome can be expected (surely more than 40,000?) to be exhaustive?
- Regarding the ePRINT application following FUS knockdown, the authors compare their data with ENCODE datasets to prove the efficiency of the method. Rather than using only published data generated in HepG2 (GSE177328) and K562 (GSE176874) cells, it would have been great to generate a CLIP dataset matching the cell line used for ePRINT (i.e. HEK293T). In addition, the peak set enrichment (figure 1J) and hypergeometric test (figure 1K) are nice representations but the exact number of targets/peaks significantly overlapping is missing. The authors may want to add those informative features for the readers.
- There are a lot of supplementary figures that could be skipped or combined to focus more on the main message of the manuscript. For example, referencing the induced mutations after UV crosslinking is a bit beyond the topic. The consequence of this widely used technic in the field for decades is quite well documented already. If the authors want to bring this kind of information, they should clearly highlight what is the novelty.

Minor points
- Finding more ePRINT peaks insides introns compared to other intragenic features is not very surprising as they represent most of the sequences in pre-mRNAs. The author should also represent the number of peaks normalized to the relative size of each feature.
- To avoid the bias toward only analyzing known motifs, it should be informative to know the proportion of ePRINT peaks without already known motifs (i.e. all peaks that the authors did not use in the motif analysis). The authors could use these populations of peaks to reveal new regulatory motifs or regions in the context of their different cell states for example. Also, how many peaks contain binding sites for multiple RBPs? How are peaks with multiple RBP motifs classified?
- The enrichment of ePRINT peaks following FUS knockdown should be represent in relation with gene expression to rule out the possibility that only highly expressed genes are analyzed here or that reduced peaks are simply down-regulated RNAs.
- The use of exoribonuclease for ePRINT should be included in the abstract as it seems to be the novelty compared to previously established methods. A sentence should be added to clearly state the advantage of XRN1 compared to other type of RNAses.
- Every panel showing a UCSC genome browser view (except Figure S2) is difficult to read and is also missing a clear y-axis scale to make sure the observed difference is not due to different scales, at least between ePRINT peaks in siNEG and siFUS.

Check again the figure legends as there are some mistakes. Examples:
- Figure S4A mentions Venn diagrams that are not present
- Figure S6 mentions the figure S2E that do not exist
- Figure S7A: UP peaks are note as reduced and enhanced as DOWN while S7B is the opposite, very misleading.

As a general comment, legends are also unequally described.
Few examples:
- Figure S1: these are not legends but a mix of methods description and interpretation of the figure
- Figure S3: What is the distinction between known and unknown variant type? How can a generated mutation be unknown?
- Figure S7A: no information about the statistical test nor the number of sequences containing each motif
- Figure S12A: number of cells not specified
- The categories of genes with "UP" and "UP only" presented in Figures 1K and S5B is very misleading. If "UP" and "DOWN" contains genes with peaks in both directions, how to make the distinctions between these two categories?
- Very minor comment: in the whole manuscript and specially in the methods section, make sure to add an unbreakable space between a number and its abbreviated units (X mM, X µL, X U, X mg/mL, …) as specified by the international convention of units.

**Reviewer 2**

Hawkins and colleagues present a new method for global profiling of RNA-protein interactions in cells or tissues: ePRINT. The authors isolate binding sites using crosslinking and exonuclease digestion and sequence resulting fragments. For HEK293T cells and motor neuron progenitors and a variety of RBP knockdown and overexpression conditions, libraries are generated, peaks are called using CLIPper, and corresponding sequences are queried for the presence of RBP motifs. The authors conclude that combining motif databases with global RNA-protein interaction profiling can efficiently answer questions in post-transcriptional gene regulation.

The work presented is a promising demonstration of the technology, although the analysis approach taken is not fully justified, and some essential details about the nature of ePRINT data are absent.

Major points
1) The authors state that CLIPper reports 41,567 peaks. This is low. CLIPper reports 26,000 peaks for AQR alone, and one would think that ePRINT would yield much more than any individual eCLIP. This calls into question whether CLIPper is an appropriate tool for ePRINT data analysis. The authors don't need to prove that CLIPper is the optimal, but it should be improving the data quality. Fig. S2 allegedly shows one example of the usefulness of size-matched input filtering but I do not understand the logic: it looks like there is a decrease in abundance with FUS knockdown, which is true with or without the size-matched input (which doesn't change) if an siRNA KD control is performed, and it is not clear that CLIPper is performing well. Relatedly, I cannot find information on the size range the authors select for in the library preparation. If a very large size range is being selected and all RNA-protein interactions are of interest, a simple approach like computing the ratio might be faster, easier, more sensitive, and more accurate.
2) The authors use a motif-centric framework to interpret the ePRINT data that while generally credible, remains an unusual choice that leaves some conspicuous unresolved points.
a. Foremost is the fact that many RBPs do not exhibit motifs that can be captured with PWMs. A more intuitive analysis would overlap CLIPper or Skipper ENCODE/ENCORE peaks with ePRINT peaks and discern what RBPs appear to contribute to ePRINT signal. RBP motifs are often not unique but may have different peak binding profiles. It would also be possible to look at peaks unique to each RBP and determine if ePRINT signal is present. If the authors have reason to believe this is not illuminating, it should be explained in the text.
b. The authors should perform some visualization or analysis showing how much ePRINT signal can be explained by individual RBPs, either with overlaps from peaks or % of peaks with motif occurrences. The motif p-value heatmap is not sufficient.
c. There appear to be over 190 RBPs with eCLIP data available on the eCLIP resource page (https://rbp-ark.com/) which suggests that the motif-centric approach is actually more limited (142 RBPs).
d. At the very least, overlap of FUS and FMR1 peaks/targets should be investigated as opposed to relying on motifs.
3) There are multiple conceptual and/or language issues with the network analysis in Figure 1M-N for motifs and CLIP data.
a. The authors write that they link "40/53 FUS KD DEGs to at least one FUS RBP" but it's not clear that the motifs are sufficiently specific to those RBPs. More broadly, the existent of a CLIP peak on a gene is not strong enough evidence to say that the RBP regulates that gene.
b. Conversely, absence of eCLIP peak evidence is not evidence of peak absence. "Analysis of published CLIP data suggested that FUS directly interacts with only 5 of these, indicating that the remaining 47 genes could be regulated by RBPs that are downstream from FUS" - the FUS Skipper output has around 30,000 windows compared to the FUS CLIPper output of around 4,500 peaks, so even the same dataset can give very different numbers of targets. If Skipper output radically changes the results it calls into question the approach employed. https://app.globus.org/file-manager?origin_id=9cd9be17-cbed-4b34-ab76-704b2fd6ac7b&origin_path=%2F
c. For some RBPs, especially with Skipper output, most expressed genes are targets, making the distinction not very meaningful. This makes the value of the analysis is generally unclear. If the links discovered are not directly functional most of the time then what are they good for? Some use case showing functional enrichment or potential for discovery by following up on nominated candidates should be included
d. Links are being discovered but there is no control process whereby random RBPs matched for the same number of peaks are chosen to label FUS KD DEGs. Without that, it's hard to know if the numbers are meaningful.
4) The paragraph starting with "Next, we utilised ePRINT data to identify genome-wide FUS binding sites" is extremely long and hard to follow. The last sentence proposing multiple motifs seems to contradict the preceding sentences showing several peaks with up- and down-regulation controlled by GU-rich elements. Co-binding or competition between RBPs could explain diverging effects for the same motif. Multiple motifs would not explain this. This and ideally other overlong paragraphs should be revised.
5) The analysis in Figure 1G is not sufficiently described. What are the background sets for the region-specific analyses? They should be matched via the input and also filtered for the same RNA region. Naturally, the intronic motifs would be enriched in introns if the same background nucleic acid sequences were being used.
6) A schematic or additions to existing panels explaining the datasets used to generate nodes and edges is needed. It doesn't help that the text uses one set of terminology (FUS-RBP) and the figures (1M-N, no current figure panel for the strategy in F2) use another (RBP with change in RNA binding activity).
7) The authors write that "multiple studies have explored the downstream targets of FUS using CLIP." That is true but they should cite the studies they are referring to and summarize relevant insights here.
8) Were Figures S9-S10 ascertained in some way or are they merely random examples? Could they be sorted according to some entropy or enrichment around 0? The takeaway is a little unclear.
9) The line "Finally, by mapping ePRINT peaks to gene features, our analysis is accurately able to predict localisation of RBPs (Fig.1G, Fig.S4B)" refers to the motif enrichment heatmaps, but I don't know what the authors mean by localization. There is no work predicting RBP occupancy presented. Indeed, the authors seem to be showing the opposite direction: that occupancy can be deconstructed into contributing RBPs.

Minor points
1) The text in Figure 2B,C is too small.
2) "As expected, increasing UV strength led to a decrease in the levels of aqueous phase RNA with an UV dose of 400 mJ/cm2 recovering ~74% of the total RNA from the interphase" - state the doses used instead of requiring viewing of the figure.
3) "We detected 204736 peaks that were identified within genes in at least two out of the four samples (Methods)." - the authors must briefly describe the methods here
4) Fig 1E: Use SNP vs. not SNP instead of known vs unknown
5) "(isolated prior to phase separation or reconstituted using protein-bound and 'free' RNA from the aqueous phase)" - which was done or which was done for which data?
6) Are the leading edge peaks in the gene ontology analysis from fgsea? This should be more detailed.
7) "Strikingly, over-expression of both candidate RBPs resulted in a significant reduction in neurite outgrowth compared to our control condition (Fig.2F, Fig.S12)." State that the control condition is overexpression of GFP in the same construct
8) "To accomplish this, we mapped the motifs of these RBPs to ePRINT peaks upregulated in MNPs vs MNs." "These RBPS" are HNRNPF and SRSF9 right? Write them out.
9) "This suggests that SRSF9 may directly regulate proteins involved in cell morphology, but also affect transcription of neuronal (or non-neuronal) genes by affecting chromatin accessibility." Gene ontology analysis is not capable of causal inference in that way. "…cell morphology or affect transcription…" is more appropriate.
10) "Our analysis approach enables global identification of RBPs with a change in activity between cell states, whereas previous methods have focused on RBP profiles on a small subset of lncRNAs in a given cell state." Are you referring to your use of DESeq2?
11) "ePRINT allows precise mapping of the 5' end of the RBP binding site, and can uncover direct and indirect targets of an RBP of interest." -> ePRINT allows precise mapping of the 5' end of the RBP binding site and uncovers direct and indirect targets of an RBP of interest
12) ", for instance, as neural progenitors differentiate into neurons." -> including neural progenitor differentiation into neurons.

**Reviewer 3**

The manuscript by Hawkins et al., presents a novel technique titled "Exonuclease assisted mapping of protein-RNA interactions (ePRINT)" which offers a new perspective on mapping RBP-RNA interaction networks on a global scale. The paper introduces a novel methodology that makes use of organic phase separation, combined with heat fragmentation and 5' end exonuclease digestion, in order to map specific RNA-bound sites in a holistic manner across the transcriptome.

Strengths:

1- The ePRINT bypasses the need to purify individual RBPs for revealing their interacting RNA regions one by one, thus greatly expanding the ability of researchers to assess RNA-RBP networks and their dynamics in a systematic manner.

2- The ePRINT procedure is very straight-forward, and does not have any highly complicated and tricky steps , thus making it easily adaptable to study of different RBP-RNA networks in diverse contexts.


Major Concerns:

1- Dependence on Prior Knowledge: The primary drawback of ePRINT is the dependence of its analysis on prior knowledge. The current analysis, in which ePRINT relies heavily on previously known data (like from CLIP studies), hinders its use for a truly unbiased assessment of RNA-RBP interaction dynamics. The authors should consider introducing unbiased approaches to analyse the resulting data, to truly unveil the method's potential. For example, they can assess if performing unbiased clustering of 5' end sequences can reveal clusters of sequences emerging from the data, that could represent similar RBP-bound motifs (irrespective of knowing the identity of these RBPs). Alternatively, structural elements that could be predicted to form at the 5' end regions could be investigated, revealing clusters of similar structures that are bound by an RBP/group of similar RBPs. Such unbiased analyses could expand the method to potentially novel RNA-RBP regulons in an unbiased way, and break from the limits of having to know the RBP binding sequences. Specially in the settings where two conditions are being compared (e.g. control and FUS KD), such unbiased sequence analyses could be focused only on the changing sequences which would be a small fraction of the total, thus reducing the data complexity and making it more likely to reveal novel changing motifs/structures.

2- Lack of Fold Enrichment Data in the Existing Motif Analyses: Another significant oversight is the omission of fold enrichment in the motif enrichment analysis figures where only p-values are reported. Understanding the depth of enrichment is vital to gauge the results' significance, especially when comparing between control and knockdown scenarios. For example, in Fig 1K, FUS binding site enrichment fold is not reported and just the p-value is. So the enrichment could be very mild (say 10% more over background). However, if FUS is depleted here, one would expect a big fold change, and not just a highly significant p-value. However, we don't know this so it is hard to properly judge these results. The same goes for several other figures (Fig 1L, Fig S5B, Fig S7B, Fig 2D, Fig S8, Fig S13).

3- Figure Organization: The organization of figures appears imbalanced. Having only two main figures contrasted with 13 supplementary figures seems inequitable and affects the manuscript's readability. Some supplementary figures could be better placed as main figures for more clarity.


Minor Concerns:

1-Readability across several figures, specifically Fig 1D, 1I, 1H, 2C, is poor. Authors need to ensure that labels and motifs are legible (some text is too small to be readable in these figures).

2- In the supplementary Fig S1A, it's imperative to display the amount of RNA from both aqueous AND interface across all UV doses. This would provide a clearer understanding of the input RNA amount, ensuring there's no loss due to UV treatment (high doses of UV can burst the cells and RNA could be therefore lost in the PBS prior to extraction. This is a well-known phenomenon and the main reason in most CLIP protocols the UV dose is restricted to 150-200 mJ). In fact, the reduction seen in the interface RNA could be not due to higher efficiency of capture, but due to a fraction being lost prior. Authors need to therefore show how much RNA they get in both phases, not just one, to demonstrate that the reduction seen is not simply because of total loss of RNA.


Recommendation:

The manuscript presents a promising method with good application potential in the RNA Biology field. However, there are key areas that need attention and modification. I suggest the authors to address the concerns raised above, before I can recommend the manuscript to be accepted for publication.

**Authors’ response**

Reviewer #1:
This study by Hawkins et al reports the development of a method, that they named ePRINT, allowing the global mapping of proteins occupancy on the transcriptome. Using UV crosslinking, organic extraction of RNA in complex with RNA-binding proteins (RBPs), and exoribonuclease treatment on fragmented RNAs, ePRINT is designed to sequence small RNA fragments originally bound by all RBP in vivo. The authors used ePRINT in HEK293T cells to map the different RBP coverage on the transcriptome with or without knockdown of FUS, an intensely studied nuclear RBP. They identified more than 40,000 peaks representing potential binding sites of all RBP in cells. A significant overlap was found between previous FUS CLIP datasets and ePRINT peaks containing FUS RNA binding motifs, suggesting that ePRINT capture true binding events. Using these data, they searched for the presence of hundreds of motifs, known to be preferentially bound by other RBPs, to identify all potential RBP with altered
RNA binding following FUS knockdown. They finally applied ePRINT during the cellular state transition of motor neurons differentiation to highlight key RBPs that could be affected in this biological context.

To develop the ePRINT method, the authors combined: (1) the recent observation that UV crosslinked RNA-RBP complexes migrate to the interphase during organic extraction, and (2) XRN1 exoribonuclease treatment of recovered RBP-RNA fragments to restrain the sequencing on the RBP footprint. In this view, the ePRINT workflow is well designed and the proper negative controls are used to remove false-positive signal. However, what contrasts their methods to existing, very similar approaches (e.g. Munschauer et al., Methods 2014 and related Schueler et al., Genome Biol., 2014) is difficult to see or at least is not enough emphasized in the manuscript. One thing that sets the ePRINT apart from previous methods is the use of XRN1 to obtain the footprint, but it is not clear whether this is advantageous as XRN1 could leave a longer 3' end on the RNA fragment compared to classical RNAses I, T1 or A. Furthermore, the authors claim that one advantage of the ePRINT is the absence of individual purification of proteins in the workflow compared to standard CLIP. That is true but at the same time a considerable amount of difficult analysis downstream of ePRINT, using known RNA binding motifs, requires the use of previous datasets established through single purification of proteins. In other words, the full analysis of ePRINT output seem highly dependent on the update of current database and on the presence of clean and interpretable CLIP datasets.

We thank the reviewer for pointing out these previous studies. These methods rely on purifying the RBP-RNA footprint using a single purification step based on nitrocellulose binding. This is insufficient to remove background RNA and hence most CLIP protocols include a step to purify the RBP-RNA complex from the membrane. Additionally, these protocols rely on over digestion of the RNA to get a higher resolution footprint (20-60 nt nt fragments). This makes mapping the reads to junctions challenging. Our method uses multiple rounds of organic extraction followed by XRN1 digestion to get rid of free RNA thus allowing a much higher signal to noise ratio. Additionally, the XRN1 digestion allows mapping the 5’ end of RNA footprint at high resolution while leaving a longer 3’end fragment. This allows precise mapping of the RNA fragment across the transcriptome including spliced junctions. Thus, we maintain resolution of the RBP footprint without compromising our mapping abilities. (pg 10)

We do not propose ePRINT as an alternative to eCLIP but as a complementary method to be used in parallel to eCLIP as demonstrated for the FUS network analysis. We have acknowledged in our article that ePRINT is reliant on high quality motifs, which can be obtained from either eCLIP or in vitro RNA BindNSeq studies. As the quality of these datasets continue to improve, this will greatly aid ePRINT analysis. Additionally, as opposed to eCLIP, ePRINT can be used to identify the downstream targets of an RBP in the absence of an IP compatible antibody. Thus, each method has its advantages and would constitute a powerful toolset when used together.

Major points
- The ePRINT output analysis presented by the authors is based on the presence of previously identified RNA-binding proteins motifs in the final obtained peaks. The conclusions that finding such motifs in ePRINT peaks relate a binding site for the associated RBP is a bit simplistic and has the main disadvantage to rely on the quality of previously established data. Hence, this analysis appears limited and biased for already well known RBPs, excluding the analysis of: (1) poorly characterized RBPs; (2) RBPs with preference for specific structures rather than sequences; (3) RBPs competing for the same motif; (4) RBPs with regulated RNA binding preferences. Applying ePRINT seems a promising tool to globally map the fraction of RBP-bound RNAs in different biological contexts but seems difficult to use for measuring the direct impact of individual RBPs if it is only based on the presence of motifs inside peaks. A key question that the author could discuss is: is finding an
enriched motif in condition A versus condition B using ePRINT necessarily means an increased binding of the associated RBP? Also, to properly perform differential analysis, ePRINT needs to be exhaustive - how many peaks per transcriptome can be expected (surely more than 40,000?) to be exhaustive?

(1) We acknowledge the reviewer’s comments that ePRINT is not a standalone method and is intended as complementary to methods such as CLIP. However, to address this dependency and explore poorly characterised RBPs, we performed an unbiased kmer analysis. We first clustered peaks based on the sequence similarity. Next, within peak clusters, we identified enrichment of known and unknown kmers (shown in Fig.1P,Q and supplementary Fig. S8) (Methods). For unknown kmers (i.e. not associated with an RBP in the Transite database), we performed GSEA to confirm that these were enriched in either up or down peaks (shown in supplementary fig S8 and supplementary files). Thus, ePRINT can be used as an investigative tool to discover novel RBP motifs. RBPs associated with these novel motifs could be identified using an in vitro experimental method similar to RNA Bind n Seq.

(2) We successfully deployed Priestess to identify secondary structure motifs associated with enriched or depleted peaks dependent on FUS knockdown (supplementary Fig. S7). Our analysis recovered motifs in different structural contexts that are similar to those identified using FUS eCLIP in addition to novel motifs. Similar to the approach described above, these analyses can be used to identify RBPs that binds these novel motifs.

(3) Though ePRINT cannot distinguish between RBPs that bind the same motif, it can be used to identify whether such motifs are differentially enriched or depleted due to a given perturbation or cell state change. This allows us to focus on a few RBPs and generate hypotheses that can be easily tested experimentally.

(4) RBPs that show a cell or perturbation specific binding can be detected using ePRINT as the corresponding motifs will display differential enrichment in the ePRINT peaks that are altered across the conditions tested. We demonstrate the utility of this approach in Fig.2 where we identified RBPs that are differentially activated as neural progenitors differentiate into neurons.

The reviewer has raised an important question: does differential enrichment of motifs indicate differential binding? Our ePRINT analysis is predicated on the hypothesis that changes in RBP target profiles are detectable through differential motif analysis in ePRINT peaks. Such changes could result from alterations in RBP expression, localization, or regulated binding, where a specific partner is needed for target location. ePRINT can identify shifts in RBP target profiles if these RBPs recognize certain motifs. However, motif enrichment doesn't always imply higher RBP binding. This discrepancy can arise from motifs hidden in secondary structures leading to alternative RBP binding, competitive binding of two RBPs to similar motifs where the RBPs display opposite binding patterns across the two conditions, and if multiple RBPs share similar motifs (pg. 9)

We agree that in principle ePRINT should identify all protein-RNA interaction events. From the eCLIP data available on ENCORE, an RBP can be assumed to have ~3000 target sites (median value of the number of targets in ENCORE eCLIP datatets (supplementary Fig. S5A). With ~1400 RBPs expressed in HEK293T cells, we estimate a maximum of 3000*1400 =~ 4 million binding events. This is of course assuming that each RBP binds independently to its target site and no sites overlap perfectly. Our reported number of binding events (41,567) is significantly lower than expected but is the result of stringent filtering of the total number of peaks identified by CLIPper. This allowed us to focus on the high confidence events and enable faster computation. By lowering the threshold, we can expand the detected binding events while still maintaining specificity as demonstrated in supplementary Fig. S5. We accept that it may not be possible to identify all binding events, but it may not be necessary to do so. The main purpose of ePRINT is to rapidly identify which binding events change and by proxy uncover which RBPs to focus on for further testing.

- Regarding the ePRINT application following FUS knockdown, the authors compare their data with ENCODE datasets to prove the efficiency of the method. Rather than using only published data generated in HepG2 (GSE177328) and K562 (GSE176874) cells, it would have been great to generate a CLIP dataset matching the cell line used for ePRINT (i.e. HEK293T). In addition, the peak set enrichment (Fig. 1J) and hypergeometric test (figure 1K) are nice representations but the exact number of targets/peaks significantly overlapping is missing. The authors may want to add those informative features for the readers.

We have successfully generated a FUS CLIP dataset in HEK293T cells and updated Fig. 1K, 1L, 1N and 1O. For the peak and gene overlaps, we have included fold enrichments over background. For the gene overlaps, we have provided the exact numbers used to calculate the hypergeometric p-values as a supplementary file to avoid adding too many numbers to the bar charts. The GSEA does not use thresholds and hence we cannot determine overlapping peak numbers for the p-value calculations. But we have reported the total number of FUS peaks that overlap with ePRINT peaks in supplementary Fig. S4B and S5C.

- There are a lot of supplementary figures that could be skipped or combined to focus more on the main message of the manuscript. For example, referencing the induced mutations after UV crosslinking is a bit beyond the topic. The consequence of this widely used technic in the field for decades is quite well documented already. If the authors want to bring this kind of information, they should clearly highlight what is the novelty.

We have removed the mutational analysis supplementary figure.

Minor points
- Finding more ePRINT peaks insides introns compared to other intragenic features is not very surprising as they represent most of the sequences in pre-mRNAs. The author should also represent the number of peaks normalized to the relative size of each feature.

We agree with the reviewer. We have included a figure (Fig. 1G) that compares the number of peaks normalized to the total length of the features. This analysis revealed that the 5’ UTRs have the highest peak density compared to all other features. We have modified the text to include this result.

- To avoid the bias toward only analyzing known motifs, it should be informative to know the proportion of ePRINT peaks without already known motifs (i.e. all peaks that the authors did not use in the motif analysis). The authors could use these populations of peaks to reveal new regulatory motifs or regions in the context of their different cell states for example. Also, how many peaks contain binding sites for multiple RBPs? How are peaks with multiple RBP motifs classified?

The majority of our peaks contain a known RBP motif (~91%). We have included a histogram in supplementary Fig. S3A that shows the number of peaks with one or more motifs. Since multiple RBPs can potentially bind to the same peak, we do not classify individual peaks in terms of RBPs. The motif analysis looks for enrichment of individual motifs across the spectrum of enriched or reduced peaks in response to a perturbation (FUS knockdown) or change in cell state (MNP to MN transition). Motifs that show differential enrichment are then used to predict the RBPs that may be involved.

- The enrichment of ePRINT peaks following FUS knockdown should be represent in relation with gene expression to rule out the possibility that only highly expressed genes are analyzed here or that reduced peaks are simply down-regulated RNAs.

This is accomplished using the expression of the gene as a co-variate in the DESeq2 model (Methods). The model considers the change in peak amplitude to the change in base gene expression to ensure that any identified differential peaks are solely due to a change in binding.

- The use of exoribonuclease for ePRINT should be included in the abstract as it seems to be the novelty compared to previously established methods. A sentence should be added to clearly state the advantage of XRN1 compared to other type of RNAses.

We have included this information in the abstract and added a sentence in the discussion as to the advantage of XRN1 (pg. 10)

- Every panel showing a UCSC genome browser view (except Figure S2) is difficult to read and is also missing a clear y-axis scale to make sure the observed difference is not due to different scales, at least between ePRINT peaks in siNEG and siFUS.

We have updated the browser figures to make these scales easier to read.

Check again the figure legends as there are some mistakes. Examples:
- Figure S4A mentions Venn diagrams that are not present: We have fixed this error.
- Figure S6 mentions the figure S2E that do not exist : We have fixed this error.
- Figure S7A: UP peaks are note as reduced and enhanced as DOWN while S7B is the opposite, very misleading: We have fixed this error and apologize for the confusion.

As a general comment, legends are also unequally described.
Few examples:
- Figure S1: these are not legends but a mix of methods description and interpretation of the figure

We have updated the legend.
- Figure S3: What is the distinction between known and unknown variant type? How can a generated mutation be unknown? – We have removed this figure. The known and unknown variants simply referred to known SNPs in the human population (independent of UV crosslink-induced mutations). We have updated the main figure labels as SNP or not SNP.
- Figure S7A: no information about the statistical test nor the number of sequences containing each motif: We have updated the figures to include this information. We realized that HOMER generates a background that is not necessarily specific to the transcriptome. Hence, we used PRIESSTESS to identify sequence-based motifs with 50000 peaks randomly selected from the transcriptome.
- Figure S12A: number of cells not specified Numbers added in figure legend
- The categories of genes with "UP" and "UP only" presented in Figures 1K and S5B is very misleading. If "UP" and "DOWN" contains genes with peaks in both directions, how to make the distinctions between these two categories?

UP indicates genes that contain a peak that is enriched while DOWN indicates genes that contain a peak that is reduced. A gene can have both UP and DOWN peaks. For example, knockdown of FUS will cause its peak to appear reduced while a compensatory binding of another RBP in a different location would be detected as an enriched peak. Such genes will be considered for both UP and DOWN overlap analysis. To confirm the specificity of the signal, the figure also includes analysis for genes that only contain UP peaks (UP only) or only contain DOWN peaks (DOWN only). Genes that display both UP and DOWN peaks were excluded for the “UP only” or “DOWN only” overlap analysis. This obviously makes the enrichment p-values less significant as many bona fide FUS targets would be excluded from the analysis as they would display both UP and DOWN peaks. This is especially apparent for the gene overlap analysis using the expanded set of ePRINT peaks.

- Very minor comment: in the whole manuscript and specially in the methods section, make sure to add an unbreakable space between a number and its abbreviated units (X mM, X µL, X U, X mg/mL, …) as specified by the international convention of units. We have made the recommended edits.

Reviewer #2: Hawkins and colleagues present a new method for global profiling of RNA-protein interactions in cells or tissues: ePRINT. The authors isolate binding sites using crosslinking and exonuclease digestion and sequence resulting fragments. For HEK293T cells and motor neuron progenitors and a variety of RBP knockdown and overexpression conditions, libraries are generated, peaks are called using CLIPper, and corresponding sequences are queried for the presence of RBP motifs. The authors conclude that combining motif databases with global RNA-protein interaction profiling can efficiently answer questions in post-transcriptional gene regulation.

The work presented is a promising demonstration of the technology, although the analysis approach taken is not fully justified, and some essential details about the nature of ePRINT data are absent.

Major points
1)      The authors state that CLIPper reports 41,567 peaks. This is low. CLIPper reports 26,000 peaks for AQR alone, and one would think that ePRINT would yield much more than any individual eCLIP. This calls into question whether CLIPper is an appropriate tool for ePRINT data analysis. The authors don't need to prove that CLIPper is the optimal, but it should be improving the data quality. Fig. S2 allegedly shows one example of the usefulness of size-matched input filtering but I do not understand the logic: it looks like there is a decrease in abundance with FUS knockdown, which is true with or without the size-matched input (which doesn't change) if an siRNA KD control is performed, and it is not clear that CLIPper is performing well. Relatedly, I cannot find information on the size range the authors select for in the library preparation. If a very large size range is being selected and all RNA-protein interactions are of interest, a simple approach like computing the ratio
might be faster, easier, more sensitive, and more accurate.

The reviewer has highlighted an important point in our analyses. The 41,567 peaks are the result of extensive filtering on the peaks identified by CLIPper. At a p-value threshold of 1e-8 and the requirement of identifying a peak in at least two out of the four samples, CLIPper identified 204,736 intragenic peaks in the FUS ePRINT datasets. We then subjected these peaks to input filtering (input enrichment significant at p < 0.001, Methods), and further filtered peaks assigned to genes with low expression (read counts < 50), resulting in the final set of 41,567 peaks.

Lowering the CLIPper threshold to 1e-4 identified 337,024 peaks. After input filtering (p < 0.01, Methods), these were reduced to 89887 intragenic peaks. Motif location, and peak and gene overlap analyses on these 89,887 peaks revealed very similar results to the original peak set i.e. most motifs are enriched within +/- 20 nucleotides from the predicted epRINT start position, there was significant overlap of FUS eCLIP peaks in the depleted ePRINT peak set and as well as at the gene level (supplementary Fig. S5).

The extensive filtering allowed us to work with peaks that had the highest confidence and keep computation times low.

We have updated supplementary Fig. S2 to include relevant examples of input filtering. The top panel in this figure provides examples of peaks that are removed during the input filtering process. These peaks are not enriched in ePRINT samples relative to input and are thus unlikely to be indicative of protein binding events. The bottom panel indicates peaks that are retained during input filtering. Peaks must be enriched in the ePRINT samples to indicate an RBP binding event. This panel also shows an example of where the size-matched input may change due to the base gene expression. Large changes in the base gene expression may artificially inflate (or mask) changes in binding events, which we account for with our design formula during differential peak analysis using DESeq2.

Libraries were selected for fragments ranging from 200 – 400 bp equivalent to RNA fragments measuring 80-280 nt. This detail has been included in the Methods (pg. 14).

2)      The authors use a motif-centric framework to interpret the ePRINT data that while generally credible, remains an unusual choice that leaves some conspicuous unresolved points.
a.      Foremost is the fact that many RBPs do not exhibit motifs that can be captured with PWMs. A more intuitive analysis would overlap CLIPper or Skipper ENCODE/ENCORE peaks with ePRINT peaks and discern what RBPs appear to contribute to ePRINT signal. RBP motifs are often not unique but may have different peak binding profiles. It would also be possible to look at peaks unique to each RBP and determine if ePRINT signal is present. If the authors have reason to believe this is not illuminating, it should be explained in the text.

We have added an explanation to this extent in the text (pg. 7). It is well documented that RBPs often target completely different RNA substrates between species and cell types, this particularly holds true for FUS and is demonstrated by the overlap of CLIP data in supplementary Fig. S4D. The motif targeted by the RBP may stay consistent however, which is why we decided to use PWMs. Multiple RBPs may match to similar motifs and would be discriminated in downstream analysis based on the goal of the end user. (an example could be by checking these peaks against CLIP data for each individual RBP).

To emphasize the flexibility of using different sources of peak information, we used the ENCODE eCLIP peaks for the GSEA as suggested by the reviewer (supplementary Fig. S6D). This analysis identified FMR1, SRSF1, SRSF7 and SRSF9 enriched in the depleted peaks, while HNRNPC was enriched in the enhanced peaks, in concordance to our analysis based on motifs. However, TIA1 was identified to be enriched in the depleted peaks as opposed to the motif analysis. This could suggest that TIA1 might identify a different set of motifs depending on specific functional contexts.

b.      The authors should perform some visualization or analysis showing how much ePRINT signal can be explained by individual RBPs, either with overlaps from peaks or % of peaks with motif occurrences. The motif p-value heatmap is not sufficient.

We have included a histogram that displays the number of peaks carrying one or multiple motifs in supplementary Fig. S3A.

c.      There appear to be over 190 RBPs with eCLIP data available on the eCLIP resource page ([https://eur03.safelinks.protection.outlook.com/?url=https%3A%2F%2Frbp-ark.com%2F&data=05%7C01%7Ca.bhinge%40exeter.ac.uk%7Cb87a6082e64d4e3c329808dbb3a911b5%7C912a5d77fb984eeeaf321334d8f04a53%7C0%7C0%7C638301310963181797%7CUnknown%7CTWFpbGZsb3d8eyJWIjoiMC4wLjAwMDAiLCJQIjoiV2luMzIiLCJBTiI6Ik1haWwiLCJXVCI6Mn0%3D%7C3000%7C%7C%7C&sdata=dqr6b%2F%2B0wYdBrPVoy4j6lVro4jVZzSccHwqbkSXrrP4%3D&reserved=0](https://eur03.safelinks.protection.outlook.com/?url=https%3A%2F%2Frbp-ark.com%2F&data=05%7C01%7CS.Hawkins3%40exeter.ac.uk%7C8dc921a9040a426959f708dbb3f41015%7C912a5d77fb984eeeaf321334d8f04a53%7C0%7C0%7C638301633090444342%7CUnknown%7CTWFpbGZsb3d8eyJWIjoiMC4wLjAwMDAiLCJQIjoiV2luMzIiLCJBTiI6Ik1haWwiLCJXVCI6Mn0%3D%7C3000%7C%7C%7C&sdata=4%2FdPXn70OgvkJywprWtggeSfRhDJC%2FvHl15S%2FjySDJA%3D&reserved=0)) which suggests that the motif-centric approach is actually more limited (142 RBPs).

We agree that the motif-centric approach appears to be limited currently but our purpose here was to demonstrate that RBP motifs can be used to discern meaningful biological insights in conjunctions with ePRINT peaks. With the inclusion of RNA BindNSeq motifs, the database of RBP motifs is expanding. Motif analysis offers an ease and flexibility of use that CLIP datasets cannot as generating CLIP data for RBPs is not trivial.

d.      At the very least, overlap of FUS and FMR1 peaks/targets should be investigated as opposed to relying on motifs.

We have shown this analysis in supplementary Fig. S6C. We found that similar to FUS eCLIP peaks, FMR1 peaks overlap significantly with ePRINT peaks that show depletion upon FUS knockdown as predicted by the motif analysis.

3)      There are multiple conceptual and/or language issues with the network analysis in Figure 1M-N for motifs and CLIP data.
a.      The authors write that they link "40/53 FUS KD DEGs to at least one FUS RBP" but it's not clear that the motifs are sufficiently specific to those RBPs. More broadly, the existent of a CLIP peak on a gene is not strong enough evidence to say that the RBP regulates that gene.

We agree that binding does not infer function necessarily but in the absence of any other evidence, this is a reasonable assumption and the basis of identifying gene targets using CLIP.

b.      Conversely, absence of eCLIP peak evidence is not evidence of peak absence. "Analysis of published CLIP data suggested that FUS directly interacts with only 5 of these, indicating that the remaining 47 genes could be regulated by RBPs that are downstream from FUS" - the FUS Skipper output has around 30,000 windows compared to the FUS CLIPper output of around 4,500 peaks, so even the same dataset can give very different numbers of targets. If Skipper output radically changes the results it calls into question the approach employed. [https://eur03.safelinks.protection.outlook.com/?url=https%3A%2F%2Fapp.globus.org%2Ffile-manager%3Forigin_id%3D9cd9be17-cbed-4b34-ab76-704b2fd6ac7b%26origin_path%3D%252F&data=05%7C01%7Ca.bhinge%40exeter.ac.uk%7Cb87a6082e64d4e3c329808dbb3a911b5%7C912a5d77fb984eeeaf321334d8f04a53%7C0%7C0%7C638301310963181797%7CUnknown%7CTWFpbGZsb3d8eyJWIjoiMC4wLjAwMDAiLCJQIjoiV2luMzIiLCJBTiI6Ik1haWwiLCJXVCI6Mn0%3D%7C3000%7C%7C%7C&sdata=eVntfQW12lutneHouId58A%2BumB8Dn%2BHaUO8sD95GhP8%3D&reserved=0](https://eur03.safelinks.protection.outlook.com/?url=https%3A%2F%2Fapp.globus.org%2Ffile-manager%3Forigin_id%3D9cd9be17-cbed-4b34-ab76-704b2fd6ac7b%26origin_path%3D%252F&data=05%7C01%7CS.Hawkins3%40exeter.ac.uk%7C8dc921a9040a426959f708dbb3f41015%7C912a5d77fb984eeeaf321334d8f04a53%7C0%7C0%7C638301633090600612%7CUnknown%7CTWFpbGZsb3d8eyJWIjoiMC4wLjAwMDAiLCJQIjoiV2luMzIiLCJBTiI6Ik1haWwiLCJXVCI6Mn0%3D%7C3000%7C%7C%7C&sdata=vcXChognI1g25U0oajCcdgNj7UpiYp1RJhnG5GgFikk%3D&reserved=0)

We generated FUS eCLIP data in HEK293T and identified ~12,000 windows mapped to hg38 genome version using Skipper. These peaks were then converted from hg38 to hg19 using the UCSC liftOver tool. After filtering for peaks with an enrichment > 2 and p-value < 0.001, we recovered 4344 peaks. These were mapped to hg19 gene annotations to retain 4121 intragenic peaks. Though we agree that absence of a FUS peak on a given RBP gene does not provide confirmatory evidence that FUS does not regulate this RBP, it is a reasonable assumption to draw in the absence of additional experimental data. Here, the main idea was to compare eCLIP and ePRINT based networks. Since the assumption remains the same across both techniques, our results remain valid.

c.      For some RBPs, especially with Skipper output, most expressed genes are targets, making the distinction not very meaningful. This makes the value of the analysis is generally unclear. If the links discovered are not directly functional most of the time then what are they good for? Some use case showing functional enrichment or potential for discovery by following up on nominated candidates should be included.

We agree that binding does not imply function. This is a problem not only relevant to ePRINT but all techniques that identify RBP binding sites including eCLIP and iCLIP. The advantage of ePRINT is that it can identify differences in the transcriptome-wide binding profiles of RBPs in response to a perturbation (FUS KD) or a change in cell state. Using a model of motor neuron progenitor differentiation, we have demonstrated that ePRINT analysis can be used to identify functional differences in RBP activity in specific cell states that we have experimentally validated (Fig 2).

d.      Links are being discovered but there is no control process whereby random RBPs matched for the same number of peaks are chosen to label FUS KD DEGs. Without that, it's hard to know if the numbers are meaningful.

We have included p-values to assess significance of our network analysis. P-values were estimated using a hypergeometric distribution as well as a random simulation. Both approaches showed that the expansion of the RBP network identified by ePRINT was statistically significant.

4)      The paragraph starting with "Next, we utilised ePRINT data to identify genome-wide FUS binding sites" is extremely long and hard to follow. The last sentence proposing multiple motifs seems to contradict the preceding sentences showing several peaks with up- and down-regulation controlled by GU-rich elements. Co-binding or competition between RBPs could explain diverging effects for the same motif. Multiple motifs would not explain this. This and ideally other overlong paragraphs should be revised.

Using a transcriptome specific background for the de novo motif analysis using PRIESTESS detected the GU-rich motif only in the reduced peak set. Hence, we have removed the earlier discussion of the same motifs being used in both the reduced and enhanced peaks. We have also simplified and segregated the paragraph to make it more comprehensible. (pg. 4,5,6)

5)      The analysis in Figure 1G is not sufficiently described. What are the background sets for the region-specific analyses? They should be matched via the input and also filtered for the same RNA region. Naturally, the intronic motifs would be enriched in introns if the same background nucleic acid sequences were being used.

Enrichment of a given motif within each feature was evaluated against the frequency of that motif across all 41,567 ePRINT peaks. Our analysis is not merely identifying generic motifs found within introns e.g. splicing acceptors or donors. To further confirm the specificity of the motifs to gene features, we evaluated the p-values with respect to a random background that was specifically generated for each gene feature. We ensured that the random background had 10 times the number of peaks in each feature as compared to ePRINT. Very similar results were obtained across the RBPs analysed (supplementary Fig. S3D). Motifs for HNRNPs, CELF3, SFPQ were enriched in introns while ELAVL3 motifs were enriched at the exon-intron junctions. SRSF and LIN28 motifs were enriched in exons but were also found to be enriched in introns. The differences observed could be because the random simulations are not enriched for regions associated with protein-binding. By using ePRINT peaks as the background, the specificity of the motifs becomes clearer.

6)      A schematic or additions to existing panels explaining the datasets used to generate nodes and edges is needed. It doesn't help that the text uses one set of terminology (FUS-RBP) and the figures (1M-N, no current figure panel for the strategy in F2) use another (RBP with change in RNA binding activity).

We have updated the figure to improve the labelling of nodes and added a short flow chart, which has been colour coded to indicate the associated nodes.

7)      The authors write that "multiple studies have explored the downstream targets of FUS using CLIP." That is true but they should cite the studies they are referring to and summarize relevant insights here.

We have included citations at this point in the text and expanded on the different functions of FUS in the preceding sentence. (pg. 3)

8)      Were Figures S9-S10 ascertained in some way or are they merely random examples? Could they be sorted according to some entropy or enrichment around 0? The takeaway is a little unclear.

These plots were originally ordered by motifs most significantly enriched in the up or down peaks, however we have re-ordered them by the enrichment z-score at position 0. The aim of these plots was to identify RBPs with a strong enrichment for the motif at position 0. It makes more sense to keep them in the order you suggest.

9)      The line "Finally, by mapping ePRINT peaks to gene features, our analysis is accurately able to predict localisation of RBPs (Fig.1G, Fig.S4B)" refers to the motif enrichment heatmaps, but I don't know what the authors mean by localization. There is no work predicting RBP occupancy presented. Indeed, the authors seem to be showing the opposite direction: that occupancy can be deconstructed into contributing RBPs.

Here, we refer to the nuclear vs cytoplasmic localization of the RBP. If an RBP motif shows enrichment specifically within introns or at exon-intron junctions, it is likely that the RBP functions within the nucleus. Alternatively, if an RBP motif displays enrichment within exons and a depletion within introns, then we can reasonably predict that the given RBP functions within the cytoplasm.

Minor points
1)      The text in Figure 2B,C is too small. We have updated all UCSC browser views to enable axis to be read. We have also increased the size of all the pie charts.
2)      "As expected, increasing UV strength led to a decrease in the levels of aqueous phase RNA with an UV dose of 400 mJ/cm2 recovering ~74% of the total RNA from the interphase" - state the doses used instead of requiring viewing of the figure. We have updated the table to include the % of RNA recovered in the aqueous phase and interphase at different UV doses.
3)      "We detected 204736 peaks that were identified within genes in at least two out of the four samples (Methods)." - the authors must briefly describe the methods here. Methods have been updated to include details on peak filtering criteria (pg 14)
4)      Fig 1E: Use SNP vs. not SNP instead of known vs unknown. Figure has been updated according to the suggestion.
5)      "(isolated prior to phase separation or reconstituted using protein-bound and 'free' RNA from the aqueous phase)" - which was done or which was done for which data? We have added the relevant information to methods
6)      Are the leading edge peaks in the gene ontology analysis from fgsea? This should be more detailed. Yes, this information is included in the motif analysis section only to avoid repetition. We have included a short note in the gene ontology section so that the reader can find this information.
7)      "Strikingly, over-expression of both candidate RBPs resulted in a significant reduction in neurite outgrowth compared to our control condition (Fig.2F, Fig.S12)." State that the control condition is overexpression of GFP in the same construct. We have added this information.
8)      "To accomplish this, we mapped the motifs of these RBPs to ePRINT peaks upregulated in MNPs vs MNs." "These RBPS" are HNRNPF and SRSF9 right? Write them out. We have added this information.
9)      "This suggests that SRSF9 may directly regulate proteins involved in cell morphology, but also affect transcription of neuronal (or non-neuronal) genes by affecting chromatin accessibility." Gene ontology analysis is not capable of causal inference in that way. "…cell morphology or affect transcription…" is more appropriate. We have edited result accordingly to read “ This suggests that SRSF9 may be involved in regulating cell morphology or chromatin accessibility.” Pg. 9
10)     "Our analysis approach enables global identification of RBPs with a change in activity between cell states, whereas previous methods have focused on RBP profiles on a small subset of lncRNAs in a given cell state." Are you referring to your use of DESeq2? No, this is referring to our more holistic approach of assessing all RBP footprints across all transcripts, rather than focus on lncRNAs or other individual RNA transcripts. We also utilise our method to uncover changes in RBP binding events between cell states, which has not been done previously.
11)     "ePRINT allows precise mapping of the 5' end of the RBP binding site, and can uncover direct and indirect targets of an RBP of interest."  -> ePRINT allows precise mapping of the 5' end of the RBP binding site and uncovers direct and indirect targets of an RBP of interest. We have updated the text as suggested.
12)     ", for instance, as neural progenitors differentiate into neurons." -> including neural progenitor differentiation into neurons. We have updated the text as suggested.

Reviewer #3: The manuscript by Hawkins et al., presents a novel technique titled "Exonuclease assisted mapping of protein-RNA interactions (ePRINT)" which offers a new perspective on mapping RBP-RNA interaction networks on a global scale. The paper introduces a novel methodology that makes use of organic phase separation, combined with heat fragmentation and 5' end exonuclease digestion, in order to map specific RNA-bound sites in a holistic manner across the transcriptome.

Strengths:

1- The ePRINT bypasses the need to purify individual RBPs for revealing their interacting RNA regions one by one, thus greatly expanding the ability of researchers to assess RNA-RBP networks and their dynamics in a systematic manner.

2- The ePRINT procedure is very straight-forward, and does not have any highly complicated and tricky steps , thus making it easily adaptable to study of different RBP-RNA networks in diverse contexts.


Major Concerns:

1- Dependence on Prior Knowledge: The primary drawback of ePRINT is the dependence of its analysis on prior knowledge. The current analysis, in which ePRINT relies heavily on previously known data (like from CLIP studies), hinders its use for a truly unbiased assessment of RNA-RBP interaction dynamics. The authors should consider introducing unbiased approaches to analyse the resulting data, to truly unveil the method's potential. For example, they can assess if performing unbiased clustering of 5' end sequences can reveal clusters of sequences emerging from the data, that could represent similar RBP-bound motifs (irrespective of knowing the identity of these RBPs). Alternatively, structural elements that could be predicted to form at the 5' end regions could be investigated, revealing clusters of similar structures that are bound by an RBP/group of similar RBPs. Such unbiased analyses could expand the method to potentially novel RNA-RBP regulons in an unbiased way, and break from the limits of having to know the RBP binding sequences. Specially in the settings where two conditions are being compared (e.g. control and FUS KD), such unbiased sequence analyses could be focused only on the changing sequences which would be a small fraction of the total, thus reducing the data complexity and making it more likely to reveal novel changing motifs/structures.

We thank the reviewer for this interesting suggestion, and agree that it would be ideal to be able to incorporate both sequence and structural information to identify RBPs of interest. We have performed the sequence clustering as suggested: we separated peaks with an increase (enhanced) or decrease (reduced) in amplitude, then scored each peak for the presence of all possible 6mers. We then clustered peaks based on these scores so that peaks that contained similar 6mer sequences were grouped together. Subsequently, we performed an enrichment analysis to identify 6mers that were enriched in each cluster vs all ePRINT peaks. We then checked whether these 6mers are linked to known RBPs, or whether these could represent unclassified RBP binding events. Our analysis recovered the established GGUG FUS and the GU-rich TDP43 motif in independent clusters in the decreased amplitude peak set. We identified 21 ‘unknown’ 6mers enriched in the reduced peak clusters and 130 ‘unknown’ 6mers in the enhanced peak clusters. Notably, many of the 6mers are similar in sequence, which could indicate activity of only a few RBPs (for instance the down clusters are biased toward c-rich sequences). (Figures 1P,Q, S8)

We deployed PRIESTESS to explore structural motifs in the enhanced or reduced ePRINT peaks and compared these results to motifs identified in the HEK293 FUS eCLIP data (supplementary Fig. S7). PRIESSTESS identified the GUGG and the related GGUG motif in an un-paired context in the FUS eCLIP dataset. Excitingly, the unpaired GUGG motif was also detected in the depleted ePRINT peaks but not in the enhanced set. This indicates that FUS binding to the GUGG sequence may occur only when the motif is present in a loop. Additionally, PRIESSTESS analysis uncovered a GGAG motif present in a hairpin loop in FUS eCLIP that was also recovered in the depleted ePRINT peak set. Due to the relative paucity of structural motif information for RBPs, we were unable to comprehensively assign the detected structural motifs to specific RBPs. We expect that further advancements in motif discovery would directly benefit ePRINT analyses. Also, using *in vitro* assays such as RNA Bind n Seq based on the identified motifs will further aid RBP identification.

2- Lack of Fold Enrichment Data in the Existing Motif Analyses: Another significant oversight is the omission of fold enrichment in the motif enrichment analysis figures where only p-values are reported. Understanding the depth of enrichment is vital to gauge the results' significance, especially when comparing between control and knockdown scenarios. For example, in Fig 1K, FUS binding site enrichment fold is not reported and just the p-value is. So the enrichment could be very mild (say 10% more over background). However, if FUS is depleted here, one would expect a big fold change, and not just a highly significant p-value. However, we don't know this so it is hard to properly judge these results. The same goes for several other figures (Fig 1L, Fig S5B, Fig S7B, Fig 2D, Fig S8, Fig S13).

We have included fold enrichments (or enrichment scores) in addition to the p-values for all gene overlap and motif analysis plots. Enrichments for the FUS direct targets discovered by eCLIP ranged from 62 % -107 % (fold enrichments of 1.67 – 2.08) when compared to ePRINT peaks depleted despite only a 50% reduction in the FUS protein.

For the GSEA analysis in supplementary Fig. S14, we have included the number of genes linked to each biological process.

3- Figure Organization: The organization of figures appears imbalanced. Having only two main figures contrasted with 13 supplementary figures seems inequitable and affects the manuscript's readability. Some supplementary figures could be better placed as main figures for more clarity.

We have removed some supplementary figures. However, since we have formatted the article as a short report, we have limited the main figures to two.

Minor Concerns:

1-Readability across several figures, specifically Fig 1D, 1I, 1H, 2C, is poor. Authors need to ensure that labels and motifs are legible (some text is too small to be readable in these figures).

All UCSC genome browser views and the relevant motif analysis figures have been updated for better readability. For Fig. 1I, we have provided a high-resolution image. This allows the large number of motifs to be discerned at higher magnification/zoom.

2- In the supplementary Fig S1A, it's imperative to display the amount of RNA from both aqueous AND interface across all UV doses. This would provide a clearer understanding of the input RNA amount, ensuring there's no loss due to UV treatment (high doses of UV can burst the cells and RNA could be therefore lost in the PBS prior to extraction. This is a well-known phenomenon and the main reason in most CLIP protocols the UV dose is restricted to 150-200 mJ). In fact, the reduction seen in the interface RNA could be not due to higher efficiency of capture, but due to a fraction being lost prior. Authors need to therefore show how much RNA they get in both phases, not just one, to demonstrate that the reduction seen is not simply because of total loss of RNA.

We repeated this experiment and did indeed identify a loss of material, suggesting that the cells were lysing during UV crosslinking. We have now reported the absolute amounts as well as the percentage of RNA captured in the aqueous phase and interphase. Our results clearly demonstrate that a depletion of RNA in the aqueous phase dependent on UV crosslinking. (supplementary Fig. S1)

Recommendation:

The manuscript presents a promising method with good application potential in the RNA Biology field. However, there are key areas that need attention and modification. I suggest the authors to address the concerns raised above, before I can recommend the manuscript to be accepted for publication.

**2^nd^ round**

**Reviewer 1**

In the revised version of the manuscript the authors comprehensively engaged with all the reviewers' concerns. I think that the manuscript clearly improved. That said, I am not convinced that the authors fully demonstrate the potential of the method to help in the interpretation of posttranscriptional gene regulatory networks, though I think that with their FUS knockdown data they have the ability to demonstrate the usefulness (and if not with FUS, perhaps adding some other RBP perturbation in their model). The authors now include FUS eCLIP data, so they now can fully relate peaks that are changing in ePRINT after FUS knockdown with FUS eCLIP peaks. And to a certain extent they are showing some analysis that demonstrates a certain enrichment of FUS eCLIP peaks with changes in ePRINT occupancy. But these analyses rely heavily on statistical methods - and I do not doubt that there is a signal. But I am wondering about the strength and reliability of the signal:
1. exactly how many FUS peaks are associated with ePRINT peaks? How many FUS peaks contain the motif and are associated with ePRINT peaks? How many FUS eCLIP peaks are associated (in absolute number; Fig. S5D offers some insight, but not absolute numbers) with ePRINT peaks that are up/down/unchanged after FUS depletion? Obviously if an ePRINT peak is unchanged it could reflect occupancy by another RBP that now has an available site, but this should be quantified in absolute numbers and discussed. How robust is the eCLIP/ePRINT relationship or does it reflect the quality of the eCLIP experiments (here it would be good to have a cleaner representation of the eCLIP experiments in the Supplement - how many mappable sequences/how many peaks/how many peaks with motif, where do these peaks distribute to, etc.

The authors acknowledge that the network analyses using ePRINT require the prior information on motifs, but they still do not use the best source of reliable RBP motifs, which are the in vitro selection procedures (RBNS foremost, but also RNAcompete). I wonder whether the network analysis would become more reliable if integrating those motifs, rather than ones discovered by the noisier eCLIP approaches.

Fig 2F may be one of the strongest arguments for the usefulness of ePRINT. Nevertheless, it remains a bit underdeveloped. E.g. authors do not rule out that the lack of neuritis outgrowth after overexpression of HNRNPF/SRSF9 may be due to off-target effects. It may have been good to overexpress some control RBP that is unregulated during differentiation (and for which the authors observe a shift in ePRINT peaks with that motif) - to rule out that transfection/manipulation is disturbing neurite outgrowth.

**Reviewer 3**

Authors have addressed all my concerns well. I am happy with the state of the revised manuscript and recommend that it is accepted.

**Authors’ response**

Reviewer #1: In the revised version of the manuscript the authors comprehensively engaged with all the reviewers' concerns. I think that the manuscript clearly improved. That said, I am not convinced that the authors fully demonstrate the potential of the method to help in the interpretation of posttranscriptional gene regulatory networks, though I think that with their FUS knockdown data they have the ability to demonstrate the usefulness (and if not with FUS, perhaps adding some other RBP perturbation in their model). The authors now include FUS eCLIP data, so they now can fully relate peaks that are changing in ePRINT after FUS knockdown with FUS eCLIP peaks. And to a certain extent they are showing some analysis that demonstrates a certain enrichment of FUS eCLIP peaks with changes in ePRINT occupancy. But these analyses rely heavily on statistical methods - and I do not doubt that there is a signal. But I am wondering about the strength and reliability of the signal:

1. exactly how many FUS peaks are associated with ePRINT peaks?

ePRINT captures ~25% of the FUS eCLIP peaks. We have reported these numbers in Fig. S4D. The percentage reported is with respect to the total number of FUS eCLIP peaks detected in HEK293 cells i.e. 4121. The thresholds used to get the final set of 4121 peaks has been included in the Methods.

2. How many FUS peaks contain the motif and are associated with ePRINT peaks?

We have reported these numbers in Figs. S4B and S4E.

3. How many FUS eCLIP peaks are associated (in absolute number; Fig. S5D offers some insight, but not absolute numbers) with ePRINT peaks that are up/down/unchanged after FUS depletion? Obviously if an ePRINT peak is unchanged it could reflect occupancy by another RBP that now has an available site, but this should be quantified in absolute numbers and discussed.

We have included the absolute numbers in Fig. S4E. As expected the number of FUS eCLIP peaks show higher overlaps with ePRINT peaks that are reduced after FUS knockdown compared with the unchanged and enhanced ePRINT peaks. However, the number of unchanged ePRINT peaks showed higher overlap with the FUS eCLIP data compared to the number of enhanced ePRINT peaks. As the reviewer has suggested, this could be because an unchanged peak can indicate a compensatory binding event by another RBP that cannot be detected by ePRINT. Regarding the motifs, we did not find any bias of the RBNS detected motifs in FUS eCLIP peaks that overlapped with the enhanced, unchanged or reduced ePRINT peaks. Pg 4 and 5.

4. How robust is the eCLIP/ePRINT relationship or does it reflect the quality of the eCLIP experiments (here it would be good to have a cleaner representation of the eCLIP experiments in the Supplement - how many mappable sequences/how many peaks/how many peaks with motif, where do these peaks distribute to, etc.

We have included mapping statistics for the FUS eCLIP data in Supplementary table 1, and the number of peaks detected in the Methods section on page 16. Fig. S4A and S4B show the number of peaks containing the canonical FUS motif. Fig S4B shows the number of FUS peaks containing the RBNS detected motifs and the associated p-values indicating string enrichment of known FUS motifs. Additionally, almost 90% of the 4121 FUS eCLIP peaks mapping within introns, in accordance with the known binding profile of FUS. Collectively, this indicates that the eCLIP data is of high quality.

The relatively low overlap of ePRINT with eCLIP peaks could be due to undersampling of the binding events in our ePRINT experiments as we have discussed on page 6.

5. The authors acknowledge that the network analyses using ePRINT require the prior information on motifs, but they still do not use the best source of reliable RBP motifs, which are the in vitro selection procedures (RBNS foremost, but also RNAcompete). I wonder whether the network analysis would become more reliable if integrating those motifs, rather than ones discovered by the noisier eCLIP approaches.

We ran the network analysis with the RBNS motifs and obtained very similar results to using the Transite motifs. The RBNS motif based network identified 41/53 DEGs (compared to 38 using transite) but lower number of network edges (244 vs 336) (Fig. S7).

6. Fig 2F may be one of the strongest arguments for the usefulness of ePRINT. Nevertheless, it remains a bit underdeveloped. E.g. authors do not rule out that the lack of neuritis outgrowth after overexpression of HNRNPF/SRSF9 may be due to off-target effects. It may have been good to overexpress some control RBP that is unregulated during differentiation (and for which the authors observe a shift in ePRINT peaks with that motif) - to rule out that transfection/manipulation is disturbing neurite outgrowth.

We acknowledge this as a valid point. However, we used the eGFP expression to control for any effects due to transduction. The three expression vectors (eGFP, SRSF9 and HNRNPF) were transfected into HEK293T cells along with the packaging plasmids at equal amounts to generate the lentiviruses. We added 0.625 ul of each virus to 10,000 progenitors in a 96-well that had 50ul of culture media. These conditions were kept the same for all three constructs. Hence, it is highly unlikely that both SRSF9 and HNRNPF overexpression caused the same off-target effect or stress that caused reduced neurite outgrowth that was not observed with the GFP lentiviral treatment.

We have included these details in the Methods section (page 18).

The use of an empty or GFP vector as a control for RBP overexpression is standard practice that is widely accepted in published peer-reviewed work as demonstrated in the articles cited below. These include papers published in Genome Biology.

https://www.nature.com/articles/s41467-020-16168-x

https://www.pnas.org/doi/10.1073/pnas.2220190120#sec-3

https://genomebiology.biomedcentral.com/articles/10.1186/s13059-020-02115-y#Sec13

https://genomebiology.biomedcentral.com/articles/10.1186/s13059-023-03149-8

https://genomebiology.biomedcentral.com/articles/10.1186/s13059-015-0771-5

<https://www.ncbi.nlm.nih.gov/pmc/articles/PMC3041167/>

If an RBP is not detected as displaying significant differential activity in our ePRINT analyses, this does not necessarily mean that the RBP is not involved in differentiation. It is possible that ePRINT may fail to detect true positives. For instance, MSI1 and MSI2 are RBPs important for the self-renewal of neural stem cells that our study did not identify. This could be because the motifs identified for these RBPs are of low quality. Additionally, the thresholds we used to identify motifs in ePRINT data might not have been ideal. We chose a relative log-odds threshold of 0.8 for identifying motifs, but this might not work best for all motifs, depending on their specificity. We suggest that using better methods based on background score distributions to determine these thresholds could lead to more accurate detection of RBPs. We have included this discussion in the main text. Pg 10.

Reviewer #3: Authors have addressed all my concerns well. I am happy with the state of the revised manuscript and recommend that it is accepted.

**3rd round**

**Reviewer 1**

The authors provided all the information that was asked of them.
Of course, I would have wished to have some of the information (particularly the obvious discrepancy between eCLIP and ePRINT) in the main figures with accompanying description. I don't consider it disqualifying that ePRINT and eCLIP only show very moderate overlap regarding changing peaks upon FUS knockdown - this is two complicated methodologies with high potential noise competing. But for the wider field to be able to use ePRINT responsibly, it would be good to have a very clear picture of the limitations.

**Authors’ response**

We have performed a direct comparison of ePRINT with eCLIP by setting hard thresholds for the ePRINT peaks. Out of the 4121 high-confidence FUS eCLIP sites identified in HEK293 cells, only 52 were proximal to at least one of the 4013 enhanced ePRINT peaks (p-adjusted < 0.05 and log2 fold change > 1), while 136 FUS sites were proximal to at least one of the 3496 reduced ePRINT peaks detected using the same thresholds (p-adjusted < 0.05 and log2 fold change < -1). Although the overlap between the reduced ePRINT peaks and FUS eCLIP sites was significant, the extent of the overlap was modest (Fig. S4E). This could be attributed to ePRINT undersampling global binding sites in addition to eCLIP detecting only a subset of the multiple closely aligned FUS binding loci. Additionally, FUS knockdown likely induces significant secondary changes in RBP profiles across the transcriptome that dominate the RBP binding landscape detected by ePRINT.

These results and discussion have been added to the main manuscript at pages 5 and 10 respectively.
